# Immunogenomic profile at baseline predicts host susceptibility to clinical malaria

**DOI:** 10.3389/fimmu.2023.1179314

**Published:** 2023-07-03

**Authors:** Gillian Mbambo, Ankit Dwivedi, Olukemi O. Ifeonu, James B. Munro, Biraj Shrestha, Robin E. Bromley, Theresa Hodges, Ricky S. Adkins, Bourema Kouriba, Issa Diarra, Amadou Niangaly, Abdoulaye K. Kone, Drissa Coulibaly, Karim Traore, Amagana Dolo, Mahamadou A. Thera, Matthew B. Laurens, Ogobara K. Doumbo, Christopher V. Plowe, Andrea A. Berry, Mark Travassos, Kirsten E. Lyke, Joana C. Silva

**Affiliations:** ^1^ Institute for Genome Sciences, University of Maryland School of Medicine, Baltimore, MD, United States; ^2^ Center for Vaccine Development and Global Health, University of Maryland School of Medicine, Baltimore, MD, United States; ^3^ Malaria Research and Training Center, International Centers for Excellence in Research (NIH), University of Science Techniques and Technologies of Bamako, Bamako, Mali; ^4^ Department of Microbiology and Immunology, University of Maryland School of Medicine, Baltimore, MD, United States; ^5^ Global Health and Tropical Medicine, Instituto deHigiene e Medicina Tropical, Universidade Nova de Lisboa (GHTM, IHMT, UNL), Lisboa, Portugal

**Keywords:** RNA sequencing, mass cytometrý, immunoinformatic analysis, baseline immunity, malaria susceptibility, malaria immunity, human parasite immunology

## Abstract

**Introduction:**

Host gene and protein expression impact susceptibility to clinical malaria, but the balance of immune cell populations, cytokines and genes that contributes to protection, remains incompletely understood. Little is known about the determinants of host susceptibility to clinical malaria at a time when acquired immunity is developing.

**Methods:**

We analyzed peripheral blood mononuclear cells (PBMCs) collected from children who differed in susceptibility to clinical malaria, all from a small town in Mali. PBMCs were collected from children aged 4-6 years at the start, peak and end of the malaria season. We characterized the immune cell composition and cytokine secretion for a subset of 20 children per timepoint (10 children with no symptomatic malaria age-matched to 10 children with >2 symptomatic malarial illnesses), and gene expression patterns for six children (three per cohort) per timepoint.

**Results:**

We observed differences between the two groups of children in the expression of genes related to cell death and inflammation; in particular, inflammatory genes such as CXCL10 and STAT1 and apoptotic genes such as XAF1 were upregulated in susceptible children before the transmission season began. We also noted higher frequency of HLA-DR+ CD4 T cells in protected children during the peak of the malaria season and comparable levels cytokine secretion after stimulation with malaria schizonts across all three time points.

**Conclusion:**

This study highlights the importance of baseline immune signatures in determining disease outcome. Our data suggests that differences in apoptotic and inflammatory gene expression patterns can serve as predictive markers of susceptibility to clinical malaria.

## Introduction

Malaria remains a major global health concern that impacts over 200 million people annually, causing an estimated 619,000 deaths in 2021 (1). Despite its global distribution throughout the tropical and subtropical regions, 95% of malaria cases occur in sub-Saharan Africa, and are caused primarily by *Plasmodium falciparum (* 1). Individuals in malaria-endemic regions with high transmission are repeatedly exposed to diverse *P. falciparum* strains and their associated antigens; these successive exposures can lead to acquired immunity to clinical malaria (2). However, children with similar levels of malaria exposure can vary in susceptibility to clinical disease. Several host factors, including those related to red blood cell disorders (HbS, HbC, G6PD and alpha-thalassemia), are known to be associated with protection, while some HLA alleles are associated with increased susceptibility to malaria infection (3, 4). Nevertheless, much remains to be understood, particularly regarding differences in the host response to infection (5).

Previous studies have implicated the activation of the type 1 interferon pathway in mitigating severe disease to mild malaria and interferon-gamma (IFNg) secretion with protection from clinical disease (6). However, other studies suggest a role for IFNg in exacerbating severe malaria episodes (cerebral malaria), implying that a delicate balance of inflammatory cytokines is critical to regulate malarial disease and pathogenesis (7). T cell-mediated immune responses play an important role in controlling pro- and anti-inflammatory immune responses during blood stage malaria (8). Type 1 helper CD4 T cells (Th1) produce inflammatory cytokines such as IFNg and TNFa that shape the early adaptive immune response to *Plasmodium* infection. These inflammatory responses are controlled by regulatory T cells (Tregs) that produce cytokines such as IL-10 and TGFb (9). Later in infection, Type 2 helper CD4 T cells (Th2) dominate and aid in antibody production, which is characteristic of a protective response to blood stage *Plasmodium* infection (10).

Transcriptomic analysis from whole blood cells has shown that naïve individuals >13 years old have a higher pro-inflammatory cytokine response when compared to their malaria-experienced counterparts upon infection with *P. falciparum (* 11). This may indicate a dampened immune response in individuals with pre-existing immunity to malaria, which could impact the clinical presentation of the disease. Some studies suggest exhausted B and T cells play a role in the lack of robust immune responses in individuals with recurrent exposure to *P. falciparum (* 12). However, it remains to be determined how molecular pathways correlate with malaria exposure history in children with distinct levels of susceptibility to clinical malaria, and which immunoprofiles characterize a protective response. Gene expression studies are increasingly used to decipher molecular mechanisms and pathways associated with malaria infection using either peripheral blood mononuclear cells (PBMCs) or whole blood (11, 13), and to analyze gene expression patterns in immune cells during malaria infection only (11, 14). Here, we compare the longitudinal transcriptional profiles of two cohorts of location and age-matched children, ages 4 to 6 years old, who differ in the degree of malaria susceptibility, to better understand the protective immune response to malaria at a time when acquired immunity is developing. We hypothesize that in an area of high malaria transmission, children who do not manifest clinical disease throughout an entire transmission season can control infection better than those who experience two or more symptomatic malaria episodes during the same period (either by preventing erythrocytic infection or by controlling symptoms), and that this difference is reflected in their PBMC transcriptomic profiles. To address this hypothesis, we compared PBMC gene expression profiles derived from a subset of children categorized as “susceptible” versus “non-susceptible/protected” to malaria, and who were part of the control arm of a malaria vaccine trial in Mali (15). An improved understanding of the host immune response to malaria, and in particular the identification of immune cell subpopulations that may play a role in a protective immune response, may identify those at increased risk and inform preventative treatment.

## Methods and sample collection

### Ethics

Sample processing was conducted with review and oversight by the University of Maryland, Baltimore’s Human Research Protection Office. The primary study was reviewed and approved by the Institutional Review Boards of the University of Maryland, Baltimore and the University of Bamako, Mali. Village permission to conduct research was obtained from village chiefs, government officials, and traditional healers prior to study initiation. Individual written informed consent was obtained from the parent or legal guardian of each child prior to screening and enrollment in accordance with the Declaration of Helsinki. Child assent was also obtained prior to study conduct.

### Study design

After obtaining informed parental consent, PBMCs from children who were part of a randomized, controlled, double-blind Apical Membrane Antigen 1 (AMA1) malaria vaccine trial in Bandiagara, Mali, West Africa, were cryopreserved on site using standardized procedures and transported to the University of Maryland, Baltimore (UMB), utilizing a tightly controlled cold chain. Registration on ClinicalTrials.gov (NCT00460525) and full study details have been previously described (15). Bandiagara, Mali, is a Sahelian town of approximately 14,000 inhabitants with highly prevalent and seasonal malaria transmission (June to December) (16, 17). There is no significant variability in the socio-economic state in this remote community. The prevalence of HIV is extremely low, and previous screening of children aged 3 months to 14 years revealed no concomitant infections with filaria. While *S. haematobium* is endemic, prevalence is typically low for children aged 2-6 years. At the time of the study conduct, children under 6 years of age routinely acquired 1-4 symptomatic malaria infections per season. All children were provided government-issued, insecticide-treated bed nets with 61% of control participants reporting use (15). Participants were continuously monitored through passive case detection and the rate of loss to follow-up was less than 7%. The Bandiagara research center served as the primary source of western medical care for most children in the village, and few had access to pharmaceuticals for intermittent malarial therapy. A subset of children (n = 25) ages 4-6 was selected from the control arm (three doses of human diploid-cell rabies vaccine (RabAvert, Chiron Vaccines)) based on age, number of clinical malaria episodes, and availability of sample across all three timepoints (15). We examined PBMCs from this cohort (**Supplementary Table 1**). The original study followed children during a single malaria transmission season (6 months) and PBMCs were collected at day 0 (beginning of transmission season), day 90 (peak transmission season), and day 150 (end of transmission season) (**Supplementary Figure 1A**). For this analysis, children with at least two clinical malaria episodes throughout the transmission season were classified as “susceptible” and were age-matched to children with no clinical malaria episodes throughout the same transmission season; the latter were classified as “protected” (**Supplementary Figure 1B**). Malaria episodes were acquired by susceptible participants between day 8 and 146. The timing of illness varied among participants, with the majority experiencing their first episode before the day 90 timepoint, except for one participant (**Supplementary Figure 1C**). Clinical malaria was defined as a symptomatic infection consistent with malaria (e.g., fever, headache, malaise) and evidence of parasitemia in the absence of an alternative clinical diagnosis. None of the children met the definition of severe malaria. Only children with PBMC available at all three time points were eligible for analysis. Serum samples were collected for each child at the same timepoints and kept at -80°C.

### Peripheral blood mononuclear cells stimulation

Cryopreserved PBMCs were thawed with fetal bovine serum (FBS) enriched media (RPMI, Gibco, Grand Island, New York) and incubated at 37°C/5% CO_2_ overnight. After incubation, PBMCs were washed and partitioned into three 1x10^6^ cell aliquots. One aliquot was stimulated with *P. falciparum* schizonts (Pfsz; derived from a Malawian source and cultured *in vitro* at UMB) at a 3:1 ratio (3 schizonts/cell) and incubated at 37°C/5% CO_2_ for 4 hours. The other two aliquots served as negative (media) and positive (stimulation with 10 mg/ml *Staphylococcus* enterotoxin B (SEB); Sigma, St. Louis, MO) controls and incubated at 37°C/5% CO_2_ for 2 hours. Golgi blockade (BD Pharmingen) was added at 0.5 mL/tube and incubated overnight (16 hours) at 37°C/5% CO_2_. As the addition of Golgi blockade compromised RNA integrity, a finding not previously noted in the literature, samples from 6 unique randomly selected children that met the age and malaria episode criteria were immediately extracted after stimulation for RNA sequencing without the addition of Golgi blockade.

### Regulatory T cell (Tregs) depletion

PBMCs were thawed as described above. A portion of the cells were partitioned to serve as negative and positive controls. The remaining PBMCs were split into two aliquots that were either mock depleted or depleted of CD25 cells using Dynabeads Pan Mouse IgG or CD25 magnetic beads, respectively (Invitrogen, Carlsbad, California) at a bead to PBMC ratio of 5:1 as described (18). PBMCS were stimulated with media, schizonts and SEB as described above.

### Staining protocol

PBMCs were stained with viability marker, Cisplatin (Pb^194/195^) (Sigma Aldrich, Indianapolis) at 1.25 mL per 500 mL for a final concentration of 25 mM. After a 1-minute incubation, PBMCs were washed with PBS supplemented with 10% fetal calf serum (FCS) then incubated with one of two optimized (13-21 cell surface markers) panels (**Supplementary Tables 2, 3**) for 20 minutes. Cells were then fixed using IC fixation buffer (eBioscience) and permeabilized using Caltag Reagent B (Invitrogen, Oregon) along with an intracellular staining cocktail (**Supplemental Tables 2, 3**), followed by the addition of DNA intercalator Iridium 191 (Ir-191 intercalator, NA). Cells were then washed 2x with cell staining media (0.2 mg/mL sodium azide in low-barium PBS supplemented with 2% FCS) and resuspended in Milli-Q water for assessment by mass cytometry. Before analysis, a viability dye, Cisplatin (19) and the DNA metalointercalator ^191/193^Ir to identify individual cells, was used to complete the panel.

### Generation of RNAseq data

Immediately after PBMC stimulation, Roche Protector RNase inhibitor (3335399001; MilliporeSigma, Burlington, Massachusetts, USA) was added to each sample. RNA was isolated using QIAzol (Qiagen 79306; Hilden, Germany) and the Direct-zol RNA Mini Prep Plus Kit (Zymo Research, Irvine, California, USA) per manufacturer’s instructions. RNA quality was assessed using the Agilent Bioanalyzer 2100, and only samples with RNA integrity numbers (RIN) greater than 7 or total RNA concentration greater than 100 ng were selected for RNA sequencing (**Supplementary Table 4**). PolyA-enriched, strand-specific RNA libraries were constructed and 100bp paired-end reads were sequenced on an Illumina NovaSeq 6000 platform. Reads were assessed for quality, trimmed if PHRED scores fell below 20, and any remaining adapter sequences were removed. These processed paired-end reads were aligned to human reference genome GRCh38 using HISAT2, and counts were generated using the HTseq analysis package (20–22). Reads that successfully mapped to the reference genome were used for downstream analyses. The data are available under bio-project ID (PRJNA603324).

### Differential gene expression analyses

To perform differential gene expression analyses, we used DEseq, EdgeR and Cuffdiff R packages (21, 23). Any genes with counts per million (CPM) values of less than 10 in 86% of the samples (or 45 of all 53 samples analyzed) were excluded from the analyses. Hemoglobin genes (the most expressed genes after those encoding rRNAs) were removed to improve power. This cutoff was selected to account for the low sample size and to ensure that each group had at least two samples present for differential expression analyses. For visualization of differentially expressed genes, normalized read counts generated by DEseq were input for the software gEAR (https://umgear.org/multigene_curator.html) to create volcano plots, implemented using the Dash Bio suite of bioinformatics components (v0.6.1 - https://github.com/plotly/dash-bio). Welch’s t-test was used to determine significance (24), and was computed using the python package diffxpy (v0.7.4 - https://github.com/theislab/diffxpy).

### Pathway and functional analysis

Pathway enrichment analysis was conducted with the output from deseq2. Differentially expressed genes with a false discovery rate (FDR) <0.05, and a log fold change (LFC) >1, were used. Genes that passed these cutoffs were submitted to DAVID web-based tool (https://david.ncifcrf.gov/tools.jsp) (25, 26). Bar plots were created with GraphPad Prism v9. Further functional analyses were performed using GSEA (27), using normalized expression values from all genes as the input.

### Mass cytometry data processing

FlowJo v.10.8.0 Software (BD Life sciences, Ashland, Oregon, USA) was used to select intact (Ir191^+^, Ir193^+^), live (PT195^-^), singlet, CD14/CD19^-^ and CD3^+^ cells. Specimens were included in the analysis if (*i*) the cell viability was >80% after thawing and (*ii*) cells were shown to be functionally active as determined by the production of IFNg by at least 0.2% CD3^+^ cells after stimulation with SEB. A response was considered specific if (*i*) the differential in the number of positive events in the stimulant pool compared to the media control was significantly increased by Chi-square analyses; and (*ii*) the net percentage of cytokine producing cells was >0.1% in stimulant pool as compared to the media control. A response was considered positive if the production of one or more cytokines, meeting the pre-defined criteria, was measured in response to antigen stimulation of PBMCs. A mean of ~200,000 cells per sample were analyzed by the CyTOF^®^ Mass Cytometer, with 71%-98% of the cells intact. After gating, CD3^+^ populations were exported *via* FlowJo to create new FCS files containing only the intact, live, CD3^+^ cell populations. FCS files were further analyzed in R version 2021.09.2 + 384 using flowCore (28) and CATALYST (29, 30) packages. FlowCore package was used to read the FCS files into R and CATALYST package was used to create a single cell experiment and transform the data with arcsinh cofactor 5. FlowSOM (31) and ConsensusClusterPlus (32) packages were used to perform high dimension clustering and generate 20 clusters using the cell surface marker panel (**Supplementary Table 2**). UMAP was used to perform high dimension reduction to visualize the clusters generated. Clusters were manually annotated based on cell surface marker expression.

### Differential analysis of mass cytometry data

Differential analysis was performed using *diffcyt* (https://bioconductor.org/packages/3.15/bioc/html/diffcyt.html), an R package that utilizes *edgeR*, *limma* and *voom* methods as part of the workflow (33). We used the *diffcyt* differential abundance (DA) method to test for differences in cell type abundance between our two study groups and the *diffcyt* differential states (DS) method to test for differences in intracellular marker expression within each identified cell population between protected and susceptible individuals. We used an FDR cutoff of <0.05 to identify differentially expressed cell types and cytokines.

### Differential antibody responses to PfEMP1 microarray

A protein microarray featuring 257 PfEMP1 protein fragments from reference and clinical infections was probed with sera collected before the malaria transmission season (day 0), from 19 children in the same cohort. Samples were randomly selected to include children whose PBMCs were processed *via* RNA sequencing and mass cytometry. Nine serum samples were from protected children while 10 serum samples were from susceptible children. Sera from a pool of North American malaria-naïve adults was used as a negative control while sera from Malian adults from the same study site collected under a different study protocol were used as positive control (34). Slide preparation and serum probing were performed as described elsewhere (35–37). Fluorescence intensity was defined as the raw signal intensity reduced by the mean for the no-DNA negative controls for each serum sample. For each protein fragment, we compared the distribution of fluorescence intensities between sera of susceptible and protected children with a two-tailed Wilcoxon rank-sum test.

## Results

### Quality control of data and experimental samples

PBMCs were processed from 26 Malian children. Cells from 6 individuals were processed through RNA sequencing and cells from 20 distinct children were profiled *via* mass cytometry. Study groups were assigned based on the number of clinical malaria episodes experienced during a single malaria transmission season.

RNAseq data was generated for six children for each of the three timepoints, and for each of the three stimulation conditions (media, schizonts and SEB), for a total of 54 samples. One SEB stimulated sample from day 150 did not pass QC and was not included in the analyses. For each sample, an average of 30 million Illumina reads were generated (**Supplementary Table 5**).

Mass cytometry data was generated for 20 different children from the same cohort. For each of the three time points, and each of the three conditions, an average of 200,000 cells were processed. Samples were functionally active if SEB stimulated controls had at least a 0.2% increase in IFNg production relative to unstimulated cells.

### Gene expression differences exist between susceptible and protected groups at baseline

**Figure 1:**
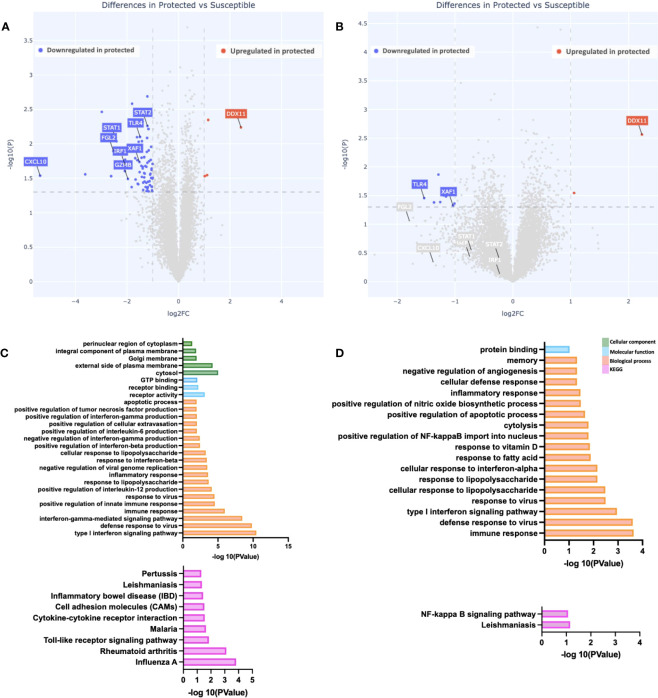
Differences in gene expression and immune pathways exist at baseline. **(A, B)** Depicted is a volcano plot of differential gene expression, with significant differences shown (blue, red), based on Welch’s Test (log10(pval) is plotted against the log2 foldchange; significance: P value <0.05). **(A)** Unstimulated PBMCs. **(B)** PBMCs stimulated with malaria antigen (*P. falciparum* schizont, Pfsz). **(C, D)** Pathway enrichment analysis on differentially expressed genes (False discovery rate, FDR<0.05; gene expression log-fold change, LFC>1), using pathway enrichment software DAVID (26). **(C)** Pathways enriched in susceptible children before antigen stimulation. **(D)** Pathways enriched in susceptible children after Pfsz stimulation. Enrichment analyses were performed on differentially expressed genes that met the FDR and LFC thresholds; no genes overexpressed in protected children met this threshold.

To assess if gene expression differences are present between malaria-protected and malaria-susceptible children, we performed a pair-wise comparison of bulk RNA sequence data to identify genes that were either significantly up-regulated or down-regulated at day 0 (start of malaria transmission season) before and after PBMC stimulation with malaria antigen. At baseline, we observed 78 genes significantly differentially expressed based on our analysis parameters (**Figure 1A**). We observed 74 downregulated and 4 upregulated genes in protected relative to susceptible children. Baseline differences in gene expression at the beginning of the malaria season suggest that differences already exist between children who later develop clinical malaria episodes and those who do not. Of interest, genes upregulated in susceptible children compared to protected children included CXCL10, STAT1, STAT2, IRF1, XAF1 and GZMB. CXCL10, STAT1 and STAT2, all genes that are downstream of interferon signaling, and IRF1, XAF1 and GZMB, which participate in apoptotic processes. Next, we evaluated gene expression patterns in Pfsz-stimulated PBMCs and observed 11 differentially expressed genes, of which 9 were downregulated and 2 upregulated in protected children relative to susceptible children. We observed some overlap in upregulated genes in susceptible children before and after antigen stimulation (**Figure 1B** and **Supplementary Figure 2A**). One gene, DDX11, was upregulated in protected children before and after Pfsz stimulation (**Figures 1A, B** and **Supplementary Figure 2B**). DDX11 encodes a helicase and plays a role in DNA repair (38). However, the direct role of this gene in regulating immune responses is not known.

To determine if the expression patterns observed were restricted to individual genes or whether, instead, they reflect broader differences at the pathway level, we searched for enriched biological, functional, and immunological pathways at day 0, with DAVID, using genes upregulated in susceptible children (FDR < 0.05; LFC >1) as input. Relative to protected children, we observed enrichment in “Type I interferon signaling,” “interferon-gamma signaling,” and “apoptotic” pathways in susceptible children (**Figures 1C, D**). Further analysis using gene set enrichment analysis (GSEA) showed enrichment in IFNg and apoptotic pathways (**Supplementary Figure 3**).

To determine whether the observed differences in gene and pathway expression were based on different levels of pre-existing immunity to malaria between the susceptible and protected groups, we probed a protein microarray populated with 257 fragments of *P. falciparum* erythrocyte membrane protein-1s (PfEMP1s) with sera from day 0 timepoint. Increased responses to PfEMP1 have been associated with recent malaria exposure (39–41). We compared the PfEMP1 serologic responses between the two groups and observed no differences in antibody responses to 99.2% of the fragments (255/257 PfEMP1 fragments; **Supplementary Figure 4B**). Additionally, we compared the proportion of individuals within each group with serorecognition of each protein fragment (defined as a fluorescence intensity greater than the mean plus two standard deviations for a serum panel from 10 malaria-naïve North American adults probed on the microarray). We observed that across extracellular PfEMP1 fragments, there was a trend towards greater proportions of protected children with serorecognition of these fragments than susceptible children [68.1% of extracellular PfEMP1 fragments(65/204)]. Interestingly, for 19 of the 20 intracellular PfEMP1 fragments on the microarray, a higher proportion of the susceptible group had serorecognition of the intracellular PfEMP1 fragment than the protected group (**Supplementary Figure 4C**). Our group previously found that serologic responses to the intracellular region of PfEMP1s is associated with greater malaria exposure (40), suggesting that the susceptible group may have had more malaria exposure than the protected group. In addition, we analyzed previously published ELISA data for Apical Membrane Antigen 1 (AMA1), another important blood stage *P. falciparum* antigen, the responses to which decrease over time since a previous clinical malaria episode (42), for individuals in the same study. A comparative analysis of 25 samples for which data was available, twelve of which were from protected and 13 of which were from susceptible children, showed no significant differences in the baseline AMA1 antibodies present in the serum collected from the children in either cohort (**Supplementary Figure 5**). The lack of differences in antibody responses to PfEMP1 and AMA1, two major blood stage malaria antigens, suggest that the differences in gene and pathway expression between groups are not due to differences in malaria exposure between the two groups. Finally, we investigated the possibility of ongoing subclinical malaria infections in susceptible children at baseline. To this effect, we analyzed previously generated data on a screen to detect the presence of parasites based on PCR amplification of the AMA1 gene from the day 0 timepoint (15). Although 2/12 individuals from the protected group were asymptomatically positive at baseline compared to 0/13 individuals from the susceptible group, we observed no statistically significant differences (Fisher’s exact test) in the percent of individuals testing positive for malaria for each study group (**Supplementary Figures 6A, B**), consistent with the hypothesis that the observed patterns are not caused by differences in ongoing or recent malaria exposure.

### Gene expression at peak malaria transmission

**Figure 2:**
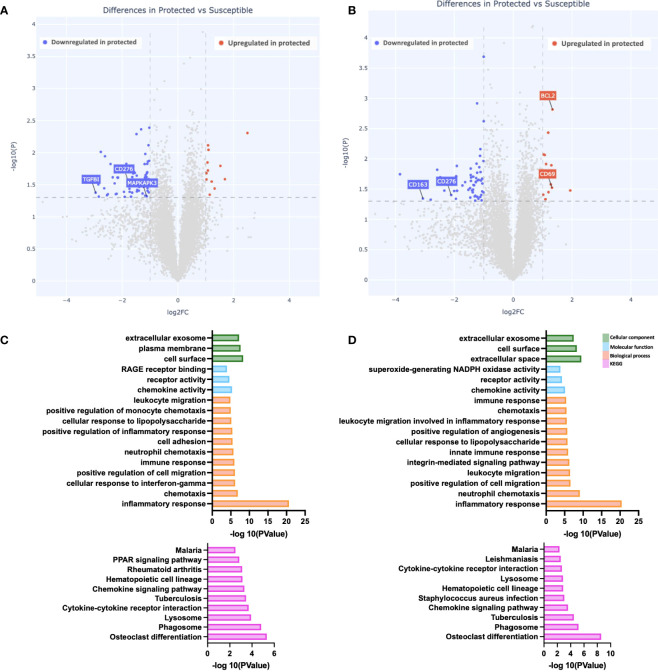
Gene expression differences during peak malaria transmission. **(A, B)** Depicted is a volcano plot of differential gene expression, with significant differences shown (blue, red), based on Welch’s Test (log10(pval) is plotted against the log2 foldchange; significance: P value <0.05). **(A)** Unstimulated PBMCs. **(B)** PBMCs stimulated with malaria antigen (*P. falciparum* schizont, Pfsz). **(C, D)** Pathway enrichment analysis on differentially expressed genes (False discovery rate, FDR<0.05; gene expression log-fold change, LFC>1), using pathway enrichment software DAVID (26). Bar graphs depict Gene Ontology; Cellular component, molecular function, and biological processes that are enriched and KEGG pathways enriched at day 90. **(C)** Pathways enriched in susceptible children before antigen stimulation. **(D)** Pathways enriched in susceptible children after Pfsz stimulation. Enrichment analyses were performed on differentially expressed genes that met the FDR and LFC thresholds.

To evaluate how increased natural exposure to malaria impacts the differences observed at baseline, we evaluated gene expression differences between the two groups at day 90 (peak malaria transmission). At this time point, we identified 73 differentially expressed genes in the unstimulated PBMCs; 61 were downregulated while 12 were upregulated in PBMCs from protected children relative to susceptible children (**Figures 2A, B**). Pathway enrichment analyses revealed significantly enriched “inflammatory response” among biological processes, in susceptible children (**Figures 2C, D**). We also observed enrichment in other biological processes in this group, including “chemotaxis” and “response to IFNg,” as well as enrichment in molecular functions including “chemokine activity” and “receptor activity” (**Figures 2C, D**). KEGG pathway analysis revealed multiple enriched pathways in susceptible children, including the “malaria” pathway (**Figures 2C, D**). Consistent with pre-stimulation observations, the biological process “inflammatory response” was significantly enriched, after Pfsz-stimulation, in susceptible children compared to protected children (**Figure 2D**).

### Reduced gene expression differences at the end of the malaria transmission season

**Figure 3:**
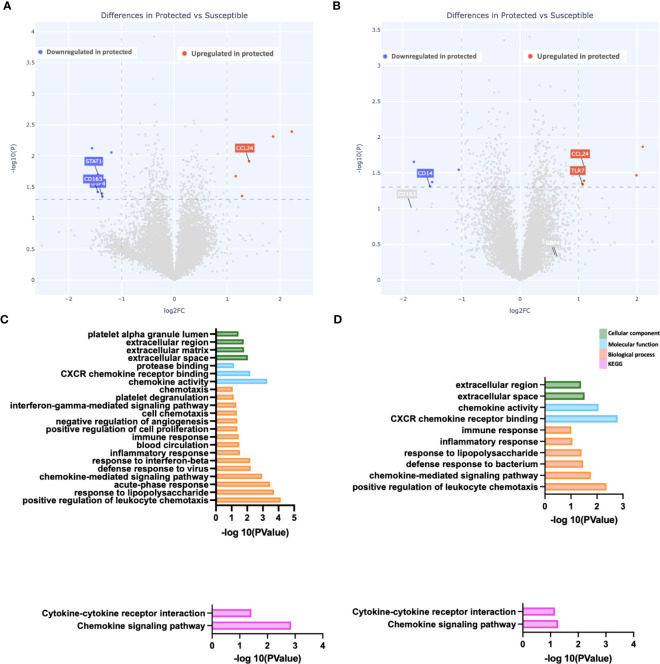
Reduced gene expression differences at the end of the malaria transmission season. **(A, B)** Depicted is a volcano plot of differential gene expression, with significant differences shown (blue, red), based on Welch’s Test (log10(pval) is plotted against the log2 foldchange; significance: P value <0.05). **(A)** Unstimulated PBMCs. **(B)** PBMCs stimulated with malaria antigen (*P. falciparum* schizont, Pfsz). **(C, D)** Pathway enrichment analysis on differentially expressed genes (False discovery rate, FDR<0.05; gene expression log-fold change, LFC>1), using pathway enrichment software DAVID (26). Bar graphs depict Gene Ontology; Cellular component, molecular function, and biological processes that are enriched and KEGG pathways enriched at day 150. **(C)** Pathways enriched in susceptible children before antigen stimulation. **(D)** Pathways enriched in susceptible children after Pfsz stimulation. Enrichment analyses were performed on differentially expressed genes that met the FDR and LFC thresholds; no genes overexpressed in protected children met this threshold.

We evaluated gene expression differences between the two groups at day 150 (end of the transmission season). We observed the fewest differences at this time point, with a total of 11 genes differentially expressed between the two groups. Six genes were downregulated compared to five genes upregulated in protected versus susceptible children (**Figure 3A**). Upregulated genes include the chemokine ligand CCL24 (**Figures 3A, B**), while downregulated genes included STAT1 and CD163 (**Figures 3A**). Pathway enrichment analysis revealed increased chemokine activity in susceptible children compared to protected children (**Figures 3C, D**). Chemokines play an important role during the host immune response to infection, as they recruit immune cells to the site of infection by binding chemokine receptors on the surface of immune cells (43). In malaria, high serum chemokine levels are associated with high parasite density and in some cases, severe malaria (44, 45).

### Similar immune cell population clustering patterns between protected and susceptible children

**Figure 4:**
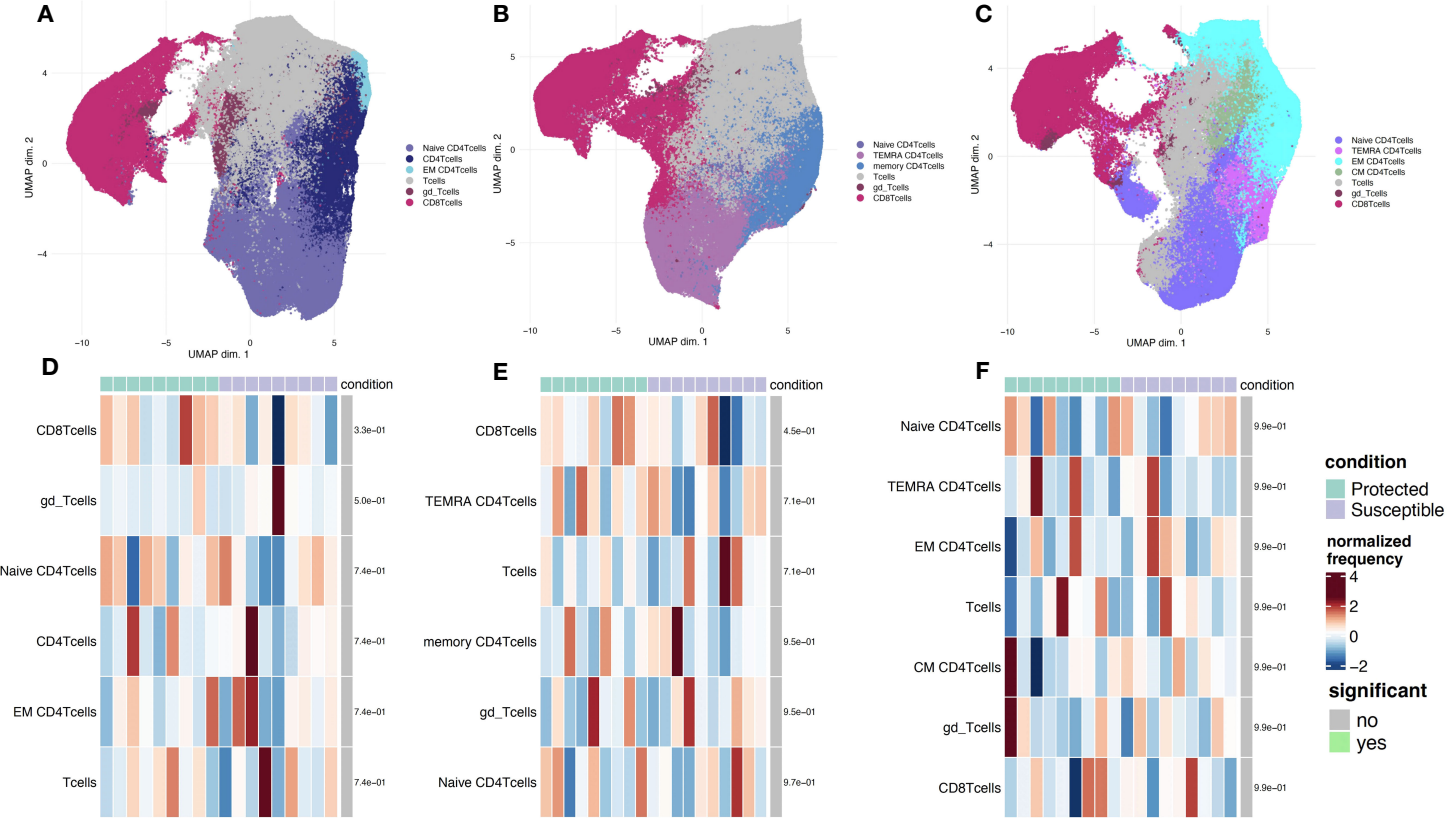
Phenotyping of CD3^+^ immune cell populations to assess effector function and cell type abundance across a single malaria transmission season. **(A-C)** UMAP plots displaying immune cell populations identified though unsupervised clustering based on cell surface marker expression. **(A)** Clustering of cells from samples collected at the day 0 timepoint. **(B)** Cells from sampled collected at day 90. **(C)** Cells from samples collected at the day 150 timepoint. **(D-F)** Heatmap showing cell type frequencies of CD3^+^ T cell populations present in protected (green) and susceptible (purple) children. High frequency populations are displayed in dark red and lowest frequencies are in dark blue. Population frequencies that are significantly different (FDR < 0.05) between protected and susceptible children are marked with a green bar. **(D)** heatmap of cell type frequencies at day 0. **(E)** Heatmap of cell type frequencies at day 90. **(F)** Heatmap of cell frequencies at day 150.

**Figure 5:**
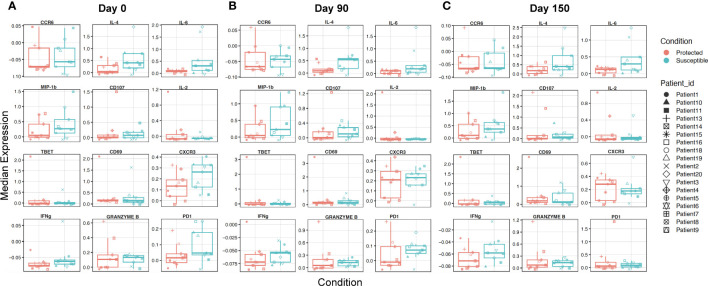
Characterizing overall expression of intracellular molecules and induced chemokine receptors in CD3^+^ cells across a single transmission season. **(A-C)** Box plots displaying overall median expression of intracellular molecules and chemokine receptors from all T cells (CD3^+^) in each Pfsz stimulated sample. Expression values were subjected to Z-score scaling, transforming the values to have a mean of 0 and a standard deviation of 1 across all cells. Negative expression values indicate that the average expression of a marker is lower relative to the other markers and positive expression values indicate that the average expression of a marker is higher relative to the other markers. Expression levels for protected children are indicated in red and expression levels for susceptible children are indicated in teal. Expression values from each child are indicated by a unique shape **(A)** Median expression for molecules at day 0. **(B)** Median expression for molecules at day 90. **(C)** Median expression for molecules at day 150.

To identify additional distinguishing properties between children with varying malaria susceptibility, we also investigated the phenotypes of immune cell populations. PBMCs were stimulated and analyzed *via* mass cytometry (see Methods). Using a panel specific for effector function CD4^+^ T cell profiling (**Supplementary Table 2**), we performed high dimensional clustering on CD3^+^ PBMCs collected at each timepoint and identified distinct cell populations (**Figures 4A-C**). Across all three timepoints, we were able to successfully identify CD8^+^, gd and naïve and memory CD4^+^ T cell populations, as well as a cluster annotated as “T cells” for cells that were not positive for either CD4^+^, CD8^+^ or gd TCR antibodies. Memory cell populations became more defined as the transmission season progressed. By day 150, we identified distinct memory CD4^+^ T cell populations, including central memory (T_CM_) (CD4^+^CD45RA^-^CCR7^+^), effector memory (T_EM_) (CD4^+^CD45RA^-^CCR7^-^), and CD45RA^+^ effector memory populations (T_EMRA_) in both protected and susceptible individuals (**Figures 4B, C**). For each identified cluster, we performed both differential cell type abundance and differential state analyses using *diffcyt* package. We observed variable cell type abundances across all individuals regardless of protected or susceptible status (**Figures 4D-F** and **Supplementary Figures 7A-F**). Differential state analysis did not detect any significant differences in intracellular marker abundance between protected and susceptible children; however, we observed slightly elevated levels of Programmed Cell Death 1 (PD-1) at day 0 and day 90 (**Figures 5A, B**), elevated IL-4 across all three timepoints (**Figures 5A-C**) and elevated IL-6 at day 150 within all PBMCs in susceptible children compared to protected children (**Figure 5C**). Within this small subset, we did not observe significant differences in cytokine production within CD4^+^ or CD8^+^ T cells alone (**Supplementary Figure 8**).

**Figure 6:**
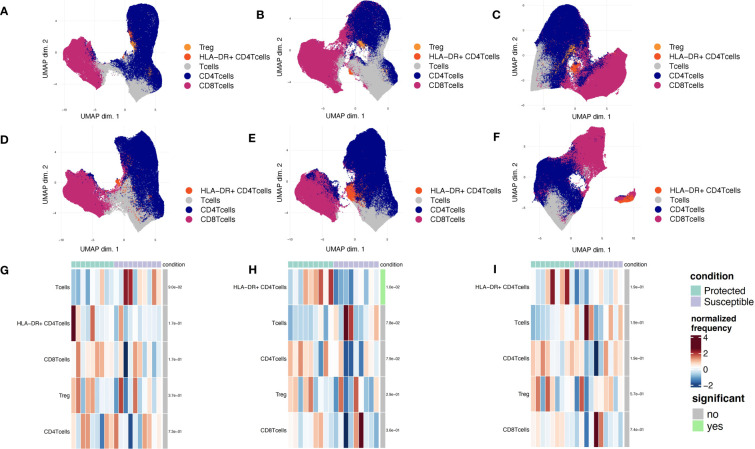
Phenotyping of CD3^+^ immune cell populations to assess regulatory functions across a single malaria transmission season. **(A-C)** UMAP plots displaying immune cell populations identified though unsupervised clustering based of PBMCs stained with the regulatory panel. **(A)** Day 0, **(B)** Day 90, and **(C)** Day 150. **(D-F)** UMAP plots displaying immune cell populations identified though unsupervised clustering of CD25-depleted PBMC stained with the regulatory panel. **(D)** Day 0, **(E)** Day 90, and **(F)** Day 150. **(G-I)** Heatmap showing cell type frequencies of CD3^+^ T cell populations present in children from each of two conditions (protected, teal; susceptible, purple). High frequency populations are displayed in dark red and lowest frequencies are in dark blue. Population frequencies that are significantly different (FDR<0.05) between protected and susceptible children are marked with a green bar (to the right of heat map). **(G)** Day 0, **(H)** Day 90, and **(I)** Day 150.

The presence of slightly elevated levels of PD1 in the mass cytometry data along with the enrichment of cell death pathways in susceptible children prompted us to investigate the role of regulatory T cells at baseline and across the transmission season. Previous studies show that the presence of Tregs may promote increased *P. falciparum* parasitemia due to a reduction in host responses to infection, and that these effects are reversed under CD25 depleted conditions (18, 46). To probe how regulatory T cells from protected and susceptible children behave as exposure to *P. falciparum* increases, we stained stimulated PBMCs with a regulatory T cell panel (**Supplementary Table 3**). We assessed cell composition and cytokine secretion patterns of stimulated CD3^+^ cells in the presence and absence of Tregs (CD4^+^CD25^+^). We were able to successfully identify a small subset of Tregs (200-1200 cells) at all three timepoints and, as expected, these cells were absent under the CD25 depleted conditions (**Figures 6A-F**). Additionally, we identified an HLA-DR^+^ CD4^+^ population across all three timepoints in both CD25^+^ present and depleted conditions (**Figures 6A-F**). HLA-DR is a human MHC class II molecule that is expressed on a variety of lymphocytes, including activated T cells (47). Differential abundance and state analyses for PBMCs stained with the regulatory panel revealed differences in the frequency of HLA-DR^+^ CD4^+^ T cells at day 90, when protected children had a higher frequency of this population relative to susceptible children (**Figures 6G-I**). We did not observe significant differences in intracellular marker expression between the two groups (**Supplementary Figure 9**). Comparison of intracellular cytokine production in PBMCs before and after CD25 depletion revealed individual-specific changes, with no significant trend noted within the entire group (**Supplementary Figures 10 A-F**). Some individuals, patients 4, 6, 9, and 10 experienced slight increase in IFNg and IL-2 levels at day 0 while patients 2, 3, and 13 experienced decrease in these cytokines at the same time point (**Supplementary Figures 10A, D**). These data demonstrate inter-individual variation present in samples collected from individuals in an endemic setting.

## Discussion

In this study, we used both bulk RNA sequencing and mass cytometry to identify host immune gene and protein expression patterns associated with varying susceptibility to clinical malaria disease. We used samples from a cohort of children who have high exposure to malaria, but who are early in the trajectory of acquiring immunity to malaria. We performed RNA sequencing on PBMCs to characterize gene expression patterns of children with different levels of malaria susceptibility. Through gene expression analyses, we identified higher baseline expression of CXCL10, STAT1, STAT2, GZMB, XAF1 and IRF1 in susceptible children relative to protected children. Our findings suggest that existing differences in gene expression, already in place before a malaria transmission season may impact susceptibility to subsequent infection. However, a new study with a larger cohort is needed to enable a study design with stronger statistical power and establish definitive conclusions. Furthermore, utilizing a regression approach can help determine the association between the number of malaria events and the enrichment of specific pathways.

Interferon gamma-induced protein 10 (CXCL10), a pro-inflammatory chemokine induced by multiple cytokines including IFNg, has been previously identified as a marker of malaria disease severity (48–50). A study of CXCL10 wildtype (WT) and Knockout (KO) mice showed efficient parasite control in KO mice while WT mice progressed to cerebral malaria (48). Additionally, a field study in Ghana found higher CXCL10 levels from postmortem cerebral spinal fluid (CSF) in cerebral malaria patients (51). Another gene downstream of IFNg signaling, STAT1, was also significantly overexpressed in the susceptible group relative to the protected group. STAT1 is a transcription factor activated by multiple interferons, and it regulates various cellular processes including cell proliferation and differentiation (52, 53). Although we did not see differential expression between groups with IFNg cytokine expression, we found evidence of gene expression differences for molecules downstream of IFN signaling, suggesting that gene expression studies may detect changes that are missed by cytokine studies.

Our pathway-level analyses revealed an enrichment of the apoptotic pathway in susceptible children. These observations are consistent with previous studies that have demonstrated increased apoptosis of immune cells (lymphopenia) in individuals with malaria infection (54, 55). Additional studies have found that malaria infection increases apoptotic processes, mediated through FAS, FASL and Tumor Necrosis Factor (TNF) (56–58). Although we did not observe increased expression of these specific genes, we observed higher expression of pro-apoptotic genes XAF1 and IRF1 in susceptible children at baseline. XAF1 is a transcriptional co-activator of IRF1, a protein that negatively regulates anti-apoptotic genes and promotes FASL expression in immune cells (59–62). The RNA-seq data were supported by mass cytometry results of slightly higher levels of PD-1 in susceptible children.

High dimension reduction and differential analysis of mass cytometry data identified higher frequencies of HLA-DR^+^CD4^+^ T cells in protected children when compared to susceptible children. HLA-DR is a late activation marker that is upregulated on the surface of either memory or naïve T cells after antigen encounter. Although the function of HLA-DR on the surface of T cells is poorly understood, studies on infectious pathogens including *Mycobacterium tuberculosis* (the causative agent of tuberculosis, or TB) and Human Immunodeficiency Virus (HIV), show that HLA-DR^+^CD4^+^ T cells have the ability to persist longer in the periphery and are more resistant to suppression mechanisms initiated by regulatory T cells (63–65).

Our data shows that inherent differences in inflammatory and apoptotic gene expression already exist at baseline between children who will go on to have two or more malaria episodes throughout a malaria season and those who will not experience clinical malaria. Recent studies report on the importance of baseline immune signatures on the subsequent response to disease progression, vaccine efficacy, and treatment failure or success (66). Tsang et al. showed a correlation between the extent of an immune response to influenza vaccination and the pre-vaccination status of an individual (67). Additionally, the importance of baseline immunity has been noted in cancer, where studies show that the presence of certain immune genes influences the activity of some immunotherapies as well as cancer metastasis (68). These studies also emphasize the importance of a systems approach to understanding overall immune signatures (66, 69).

Our current study corroborates and extends previous findings. We compared children with two or more episodes of clinical malaria (susceptible cohort) to age- and location-matched children without clinical malaria (protected cohort). Although the cohorts had similar risk of malaria exposure, we identified differences in gene expression patterns assessed before the onset of the malaria transmission season between the cohorts. This study emphasizes the importance of understanding the initial gene expression repertoire to accurately interpret clinical and immunological implications of field studies with human subjects. In conclusion, this study identifies potential genes and cell populations associated with, and which may play a causal role in, predicting malaria susceptibility.

## Data availability statement

The datasets presented in this study can be found in online repositories. The names of the repository/repositories and accession number(s) can be found below: PRJNA603324 (Bioproject- Genbank) and GEO Accession GSE234970.

## Ethics statement

The studies involving human participants were reviewed and approved by Institutional Review Board for Human Subject Research at University of Maryland Baltimore. Written informed consent to participate in this study was provided by the participants’ legal guardian/next of kin.

## Author contributions

GM, JS, and KL were involved in study design and execution of current study. KL, ML, CP, and MAT were involved in the execution of the primary study. GM, KL, and RB contributed to protocol optimization. GM, AnD, and OOI contributed to bioinformatics analysis. TH and RA contributed to software optimization and training. MAT, OKD, AK, DC, and AB were responsible for study management in Mali. BS, AB, and MT contributed to microarray data generation and analysis. GM, JS, and KL contributed to interpretation and writing of the article. All authors participated in editing and approved the submitted version.
