# Supplementary material for: Immunogenomic profile at baseline predicts host susceptibility to clinical malaria
Source: Front Immunol. 2023 Jul 3;14:1179314. doi: 10.3389/fimmu.2023.1179314 (PMC10351378; doi:10.3389/fimmu.2023.1179314)
Supplement: Supplementary file 1 [file DataSheet_1.zip › supplementary_material/supplementary_table_1.docx]

| **Volunteer** | **Group** | **Assay** | **Age** | **Ethnicity** | **Sex** | **# of Pf events** |
| --- | --- | --- | --- | --- | --- | --- |
| Patient 1 | Protected | Mass Cytometry/Array | 5.24 | Peulh | M | 0 |
| Patient 4 | Protected | Mass Cytometry/Array | 4.87 | Dogon | F | 0 |
| Patient 6 | Protected | Mass Cytometry/Array | 5.81 | Bambara | M | 0 |
| Patient 9 | Protected | Mass Cytometry | 2.52 | Dogon | F | 0 |
| Patient 11 | Protected | Mass Cytometry/Array | 5.85 | Bambara | F | 0 |
| Patient 13 | Protected | Mass Cytometry/Array | 4.8 | Dogon | M | 0 |
| Patient 14 | Protected | Mass Cytometry | 6.78 | Dogon | F | 0 |
| Patient 15 | Protected | Mass Cytometry/Array | 4.09 | Sonike | M | 0 |
| Patient 18 | Protected | Mass Cytometry/Array | 5.05 | Dogon | F | 0 |
| Patient 21 | Protected | RNA-seq | 6.74 | Dogon | F | 0 |
| Patient 22 | Protected | RNA-seq/Array | 4.39 | Dogon | F | 0 |
| Patient 23 | Protected | RNA-seq/Array | 6.32 | Dogon | F | 0 |
| Patient 2 | Susceptible | Mass Cytometry | 4.88 | Dogon | F | 3 |
| Patient 3 | Susceptible | Mass Cytometry | 5.78 | Dogon | M | 2 |
| Patient 5 | Susceptible | Mass Cytometry/Array | 5.9 | Dogon | M | 3 |
| Patient 7 | Susceptible | Mass Cytometry/Array | 6.04 | Dogon | M | 3 |
| Patient 8 | Susceptible | Mass Cytometry/Array | 5.22 | Dogon | M | 2 |
| Patient 10 | Susceptible | Mass Cytometry/Array | 6.77 | Dogon | F | 4 |
| Patient 12 | Susceptible | Mass Cytometry/Array | 4.02 | Dogon | F | 3 |
| Patient 16 | Susceptible | Mass Cytometry | 4.11 | Dogon | F | 2 |
| Patient 19 | Susceptible | Mass Cytometry/Array | 5.99 | Dogon | M | 2 |
| Patient 20 | Susceptible | Mass Cytometry | 4.04 | Dogon | F | 2 |
| Patient 24 | Susceptible | RNA-seq/Array | 6.0 | Dogon | M | 2 |
| Patient 25 | Susceptible | RNA-seq | 6.09 | Dogon | F | 2 |
| Patient 26 | Susceptible | RNA-seq | 4.81 | Dogon | F | 2 |

**Supplementary Table 1: Cohort Demographics.** Table displaying participant ID along with their assigned group condition (protected or susceptible), assay performed, age, sex, ethinicity, and the number of clinical malaria episodes, experienced by each individual.
