# Supplementary material for: Immunogenomic profile at baseline predicts host susceptibility to clinical malaria
Source: Front Immunol. 2023 Jul 3;14:1179314. doi: 10.3389/fimmu.2023.1179314 (PMC10351378; doi:10.3389/fimmu.2023.1179314)
Supplement: Supplementary file 1 [file DataSheet_1.zip › supplementary_material/supplemental_figures.pptx]

## Slide 1
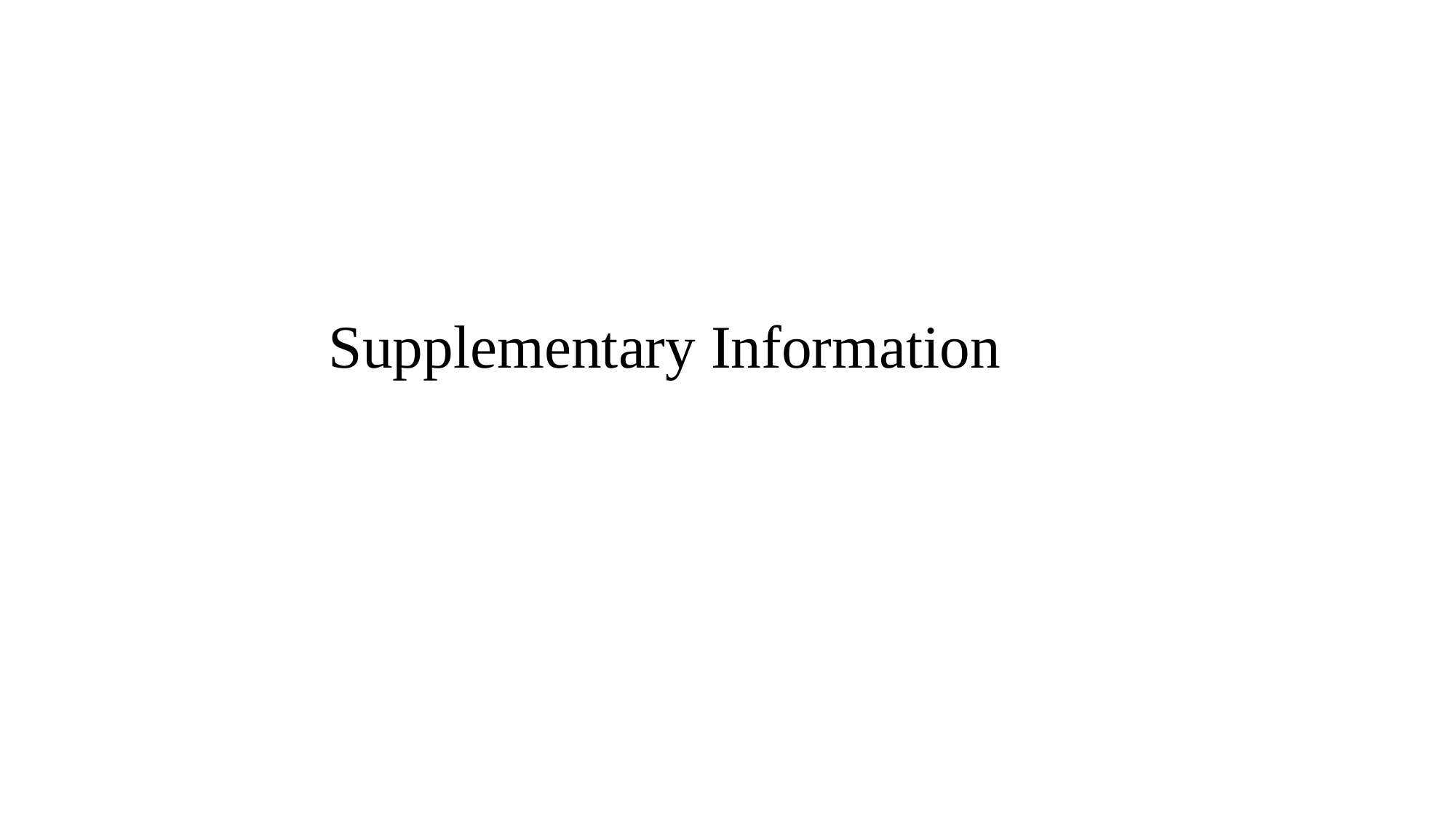

# Supplementary Information

## Slide 2
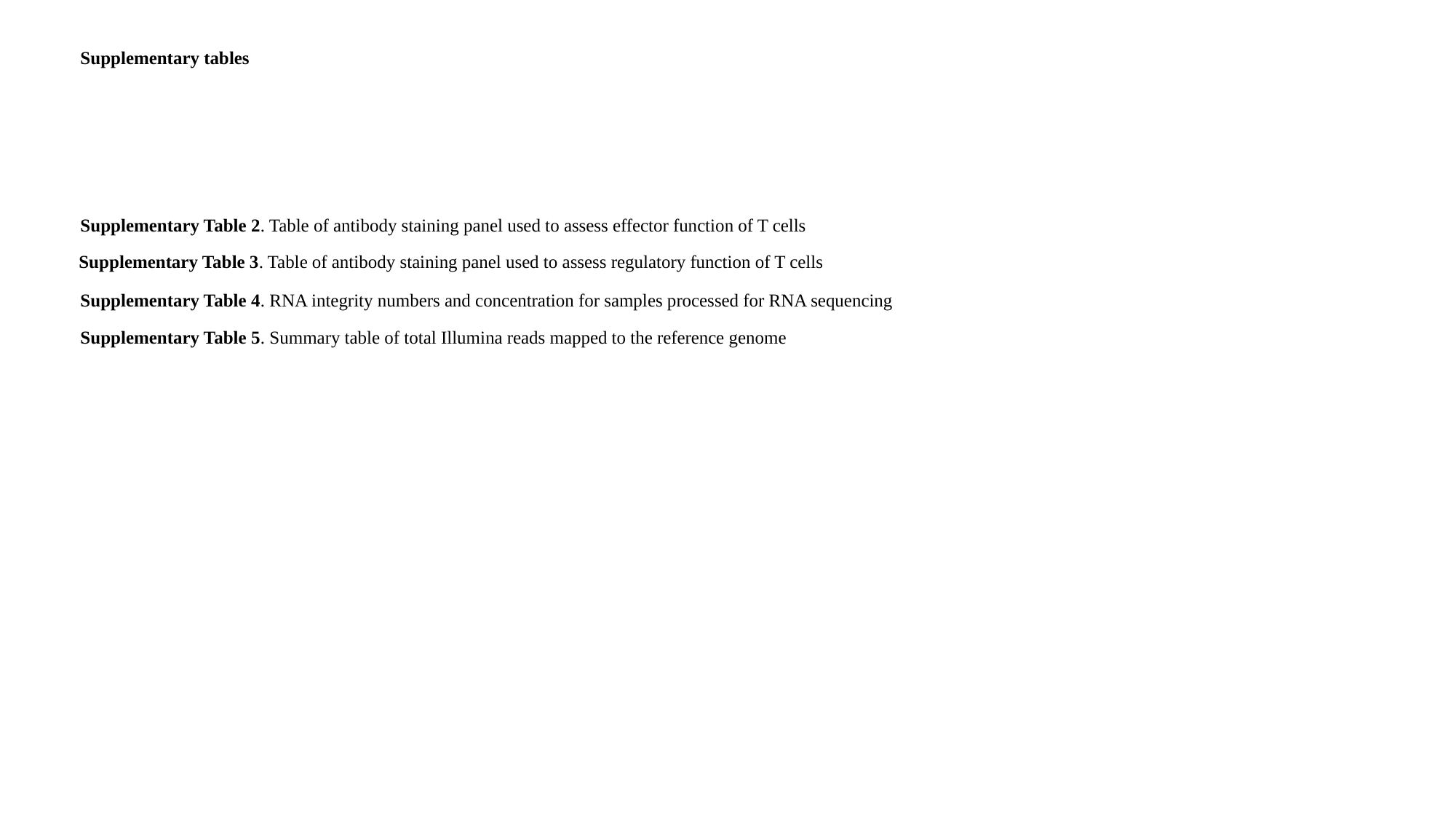

# Supplementary tables
Supplementary Table 2. Table of antibody staining panel used to assess effector function of T cells
Supplementary Table 3. Table of antibody staining panel used to assess regulatory function of T cells
Supplementary Table 4. RNA integrity numbers and concentration for samples processed for RNA sequencing
Supplementary Table 5. Summary table of total Illumina reads mapped to the reference genome

## Slide 3
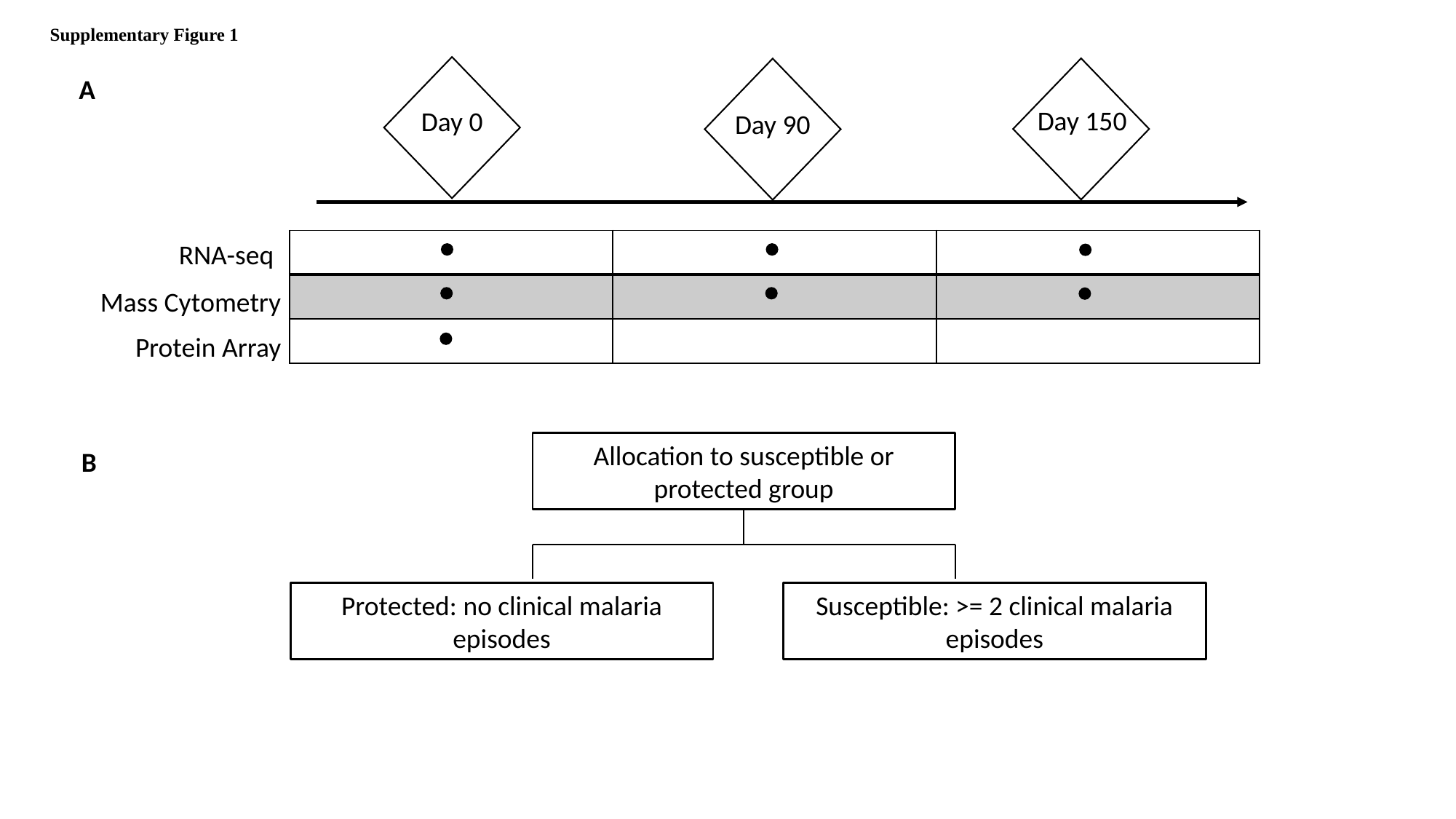

# Supplementary Figure 1
A
Day 150
Day 0
Day 90
| | | |
| --- | --- | --- |
| | | |
| | | |
RNA-seq
Mass Cytometry
Protein Array
Allocation to susceptible or protected group
B
Protected: no clinical malaria episodes
Susceptible: >= 2 clinical malaria episodes

## Slide 4
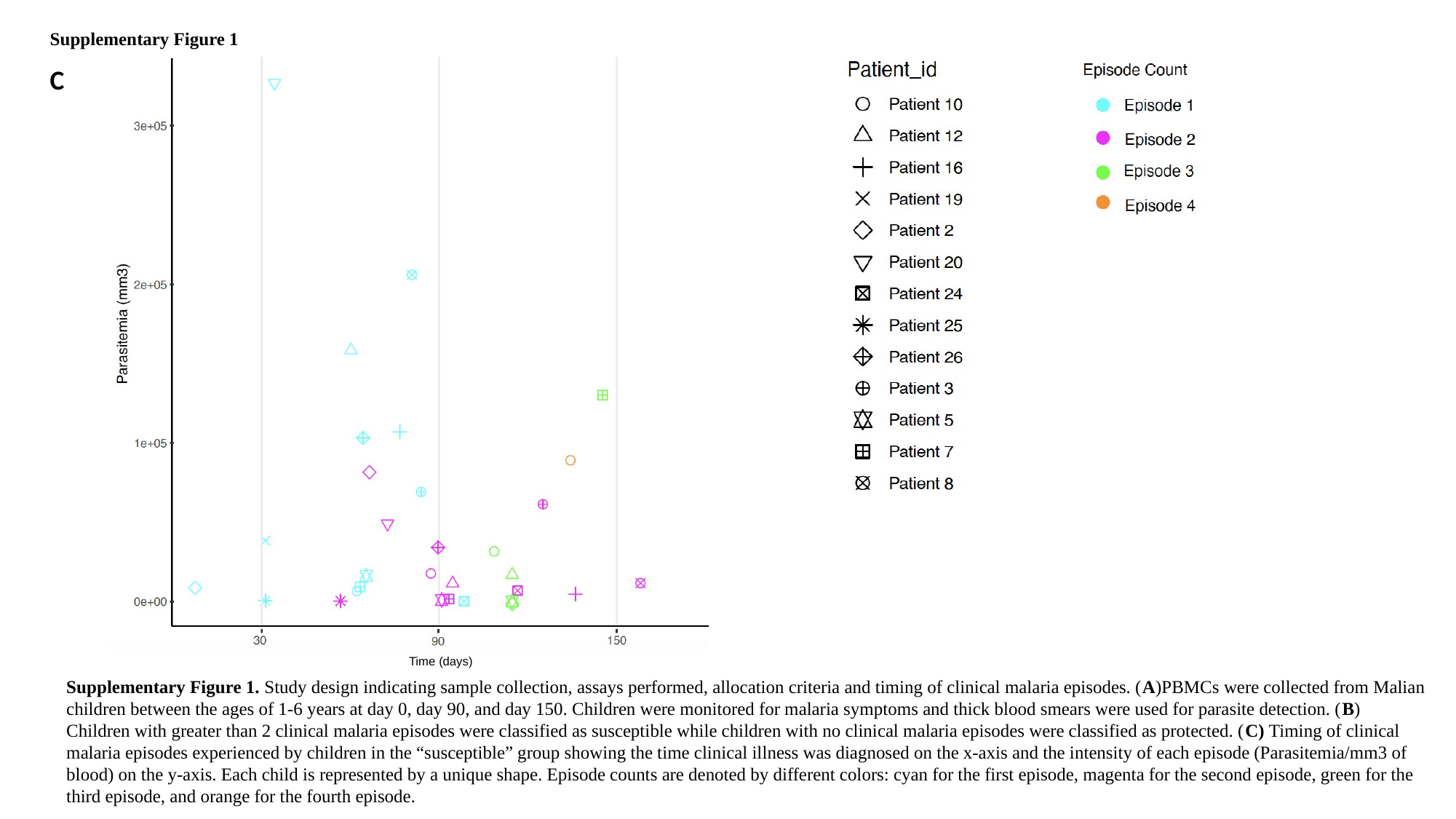

# Supplementary Figure 1
C
Time (days)
Supplementary Figure 1. Study design indicating sample collection, assays performed, allocation criteria and timing of clinical malaria episodes. (A)PBMCs were collected from Malian children between the ages of 1-6 years at day 0, day 90, and day 150. Children were monitored for malaria symptoms and thick blood smears were used for parasite detection. (B) Children with greater than 2 clinical malaria episodes were classified as susceptible while children with no clinical malaria episodes were classified as protected. (C) Timing of clinical malaria episodes experienced by children in the “susceptible” group showing the time clinical illness was diagnosed on the x-axis and the intensity of each episode (Parasitemia/mm3 of blood) on the y-axis. Each child is represented by a unique shape. Episode counts are denoted by different colors: cyan for the first episode, magenta for the second episode, green for the third episode, and orange for the fourth episode.

## Slide 5
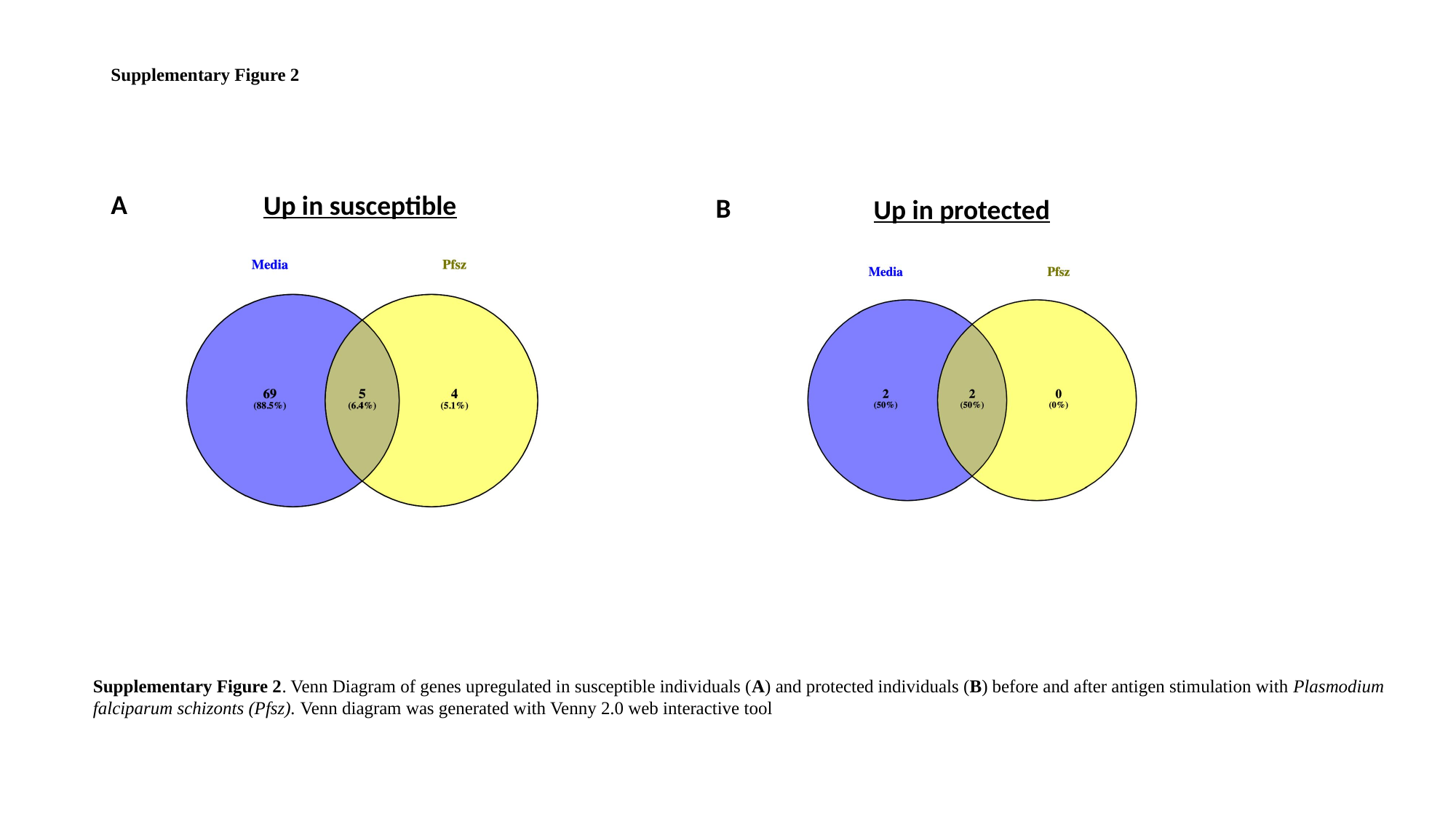

# Supplementary Figure 2
A
Up in susceptible
B
Up in protected
Supplementary Figure 2. Venn Diagram of genes upregulated in susceptible individuals (A) and protected individuals (B) before and after antigen stimulation with Plasmodium falciparum schizonts (Pfsz). Venn diagram was generated with Venny 2.0 web interactive tool

## Slide 6
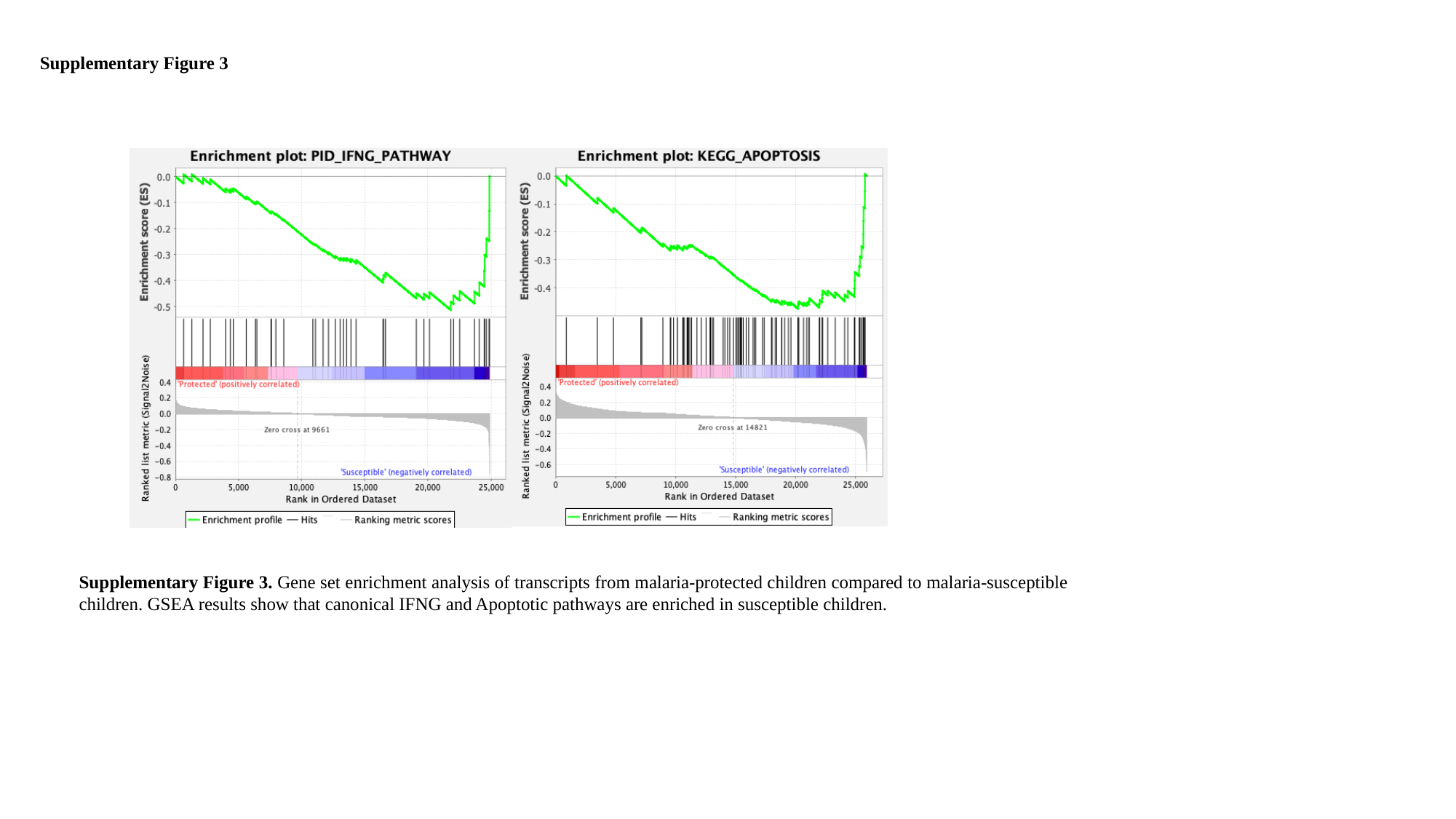

# Supplementary Figure 3
Supplementary Figure 3. Gene set enrichment analysis of transcripts from malaria-protected children compared to malaria-susceptible children. GSEA results show that canonical IFNG and Apoptotic pathways are enriched in susceptible children.

## Slide 7
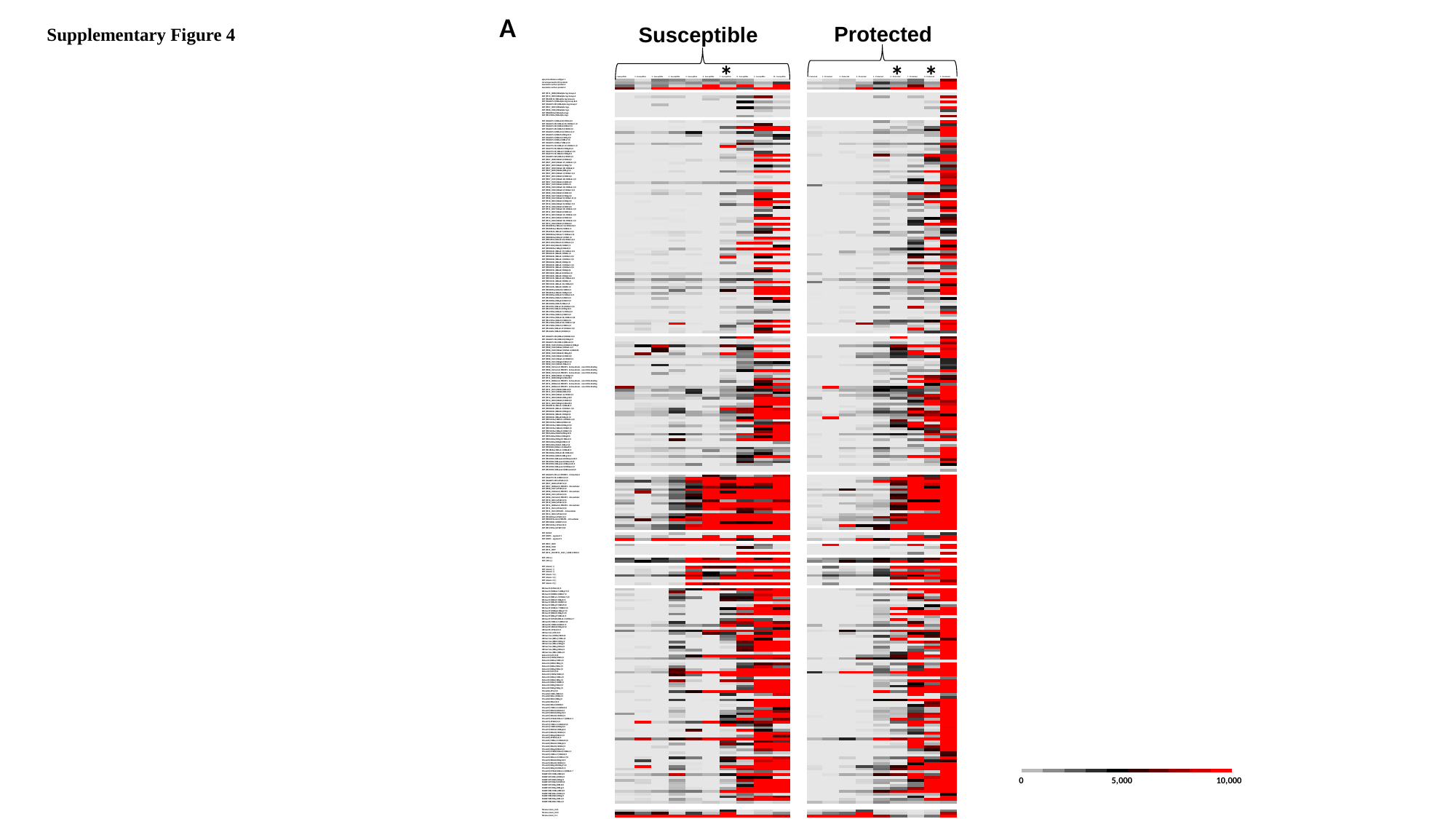

A
Supplementary Figure 4
Protected
Susceptible
*
*
*
0 5,000 10,000

## Slide 8
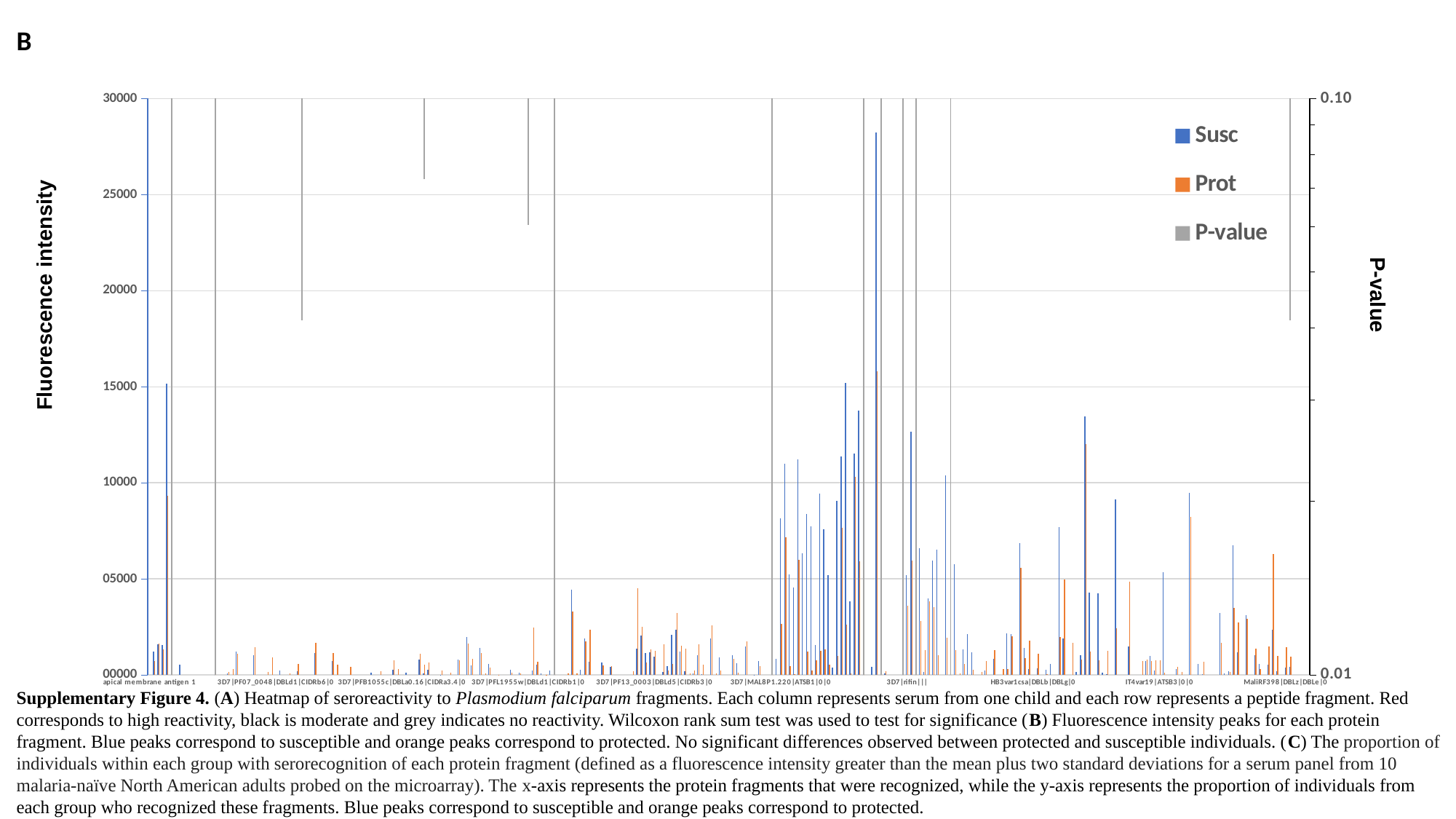

B
[unsupported chart]
Fluorescence intensity
P-value
Supplementary Figure 4. (A) Heatmap of seroreactivity to Plasmodium falciparum fragments. Each column represents serum from one child and each row represents a peptide fragment. Red corresponds to high reactivity, black is moderate and grey indicates no reactivity. Wilcoxon rank sum test was used to test for significance (B) Fluorescence intensity peaks for each protein fragment. Blue peaks correspond to susceptible and orange peaks correspond to protected. No significant differences observed between protected and susceptible individuals. (C) The proportion of individuals within each group with serorecognition of each protein fragment (defined as a fluorescence intensity greater than the mean plus two standard deviations for a serum panel from 10 malaria-naïve North American adults probed on the microarray). The x-axis represents the protein fragments that were recognized, while the y-axis represents the proportion of individuals from each group who recognized these fragments. Blue peaks correspond to susceptible and orange peaks correspond to protected.

## Slide 9
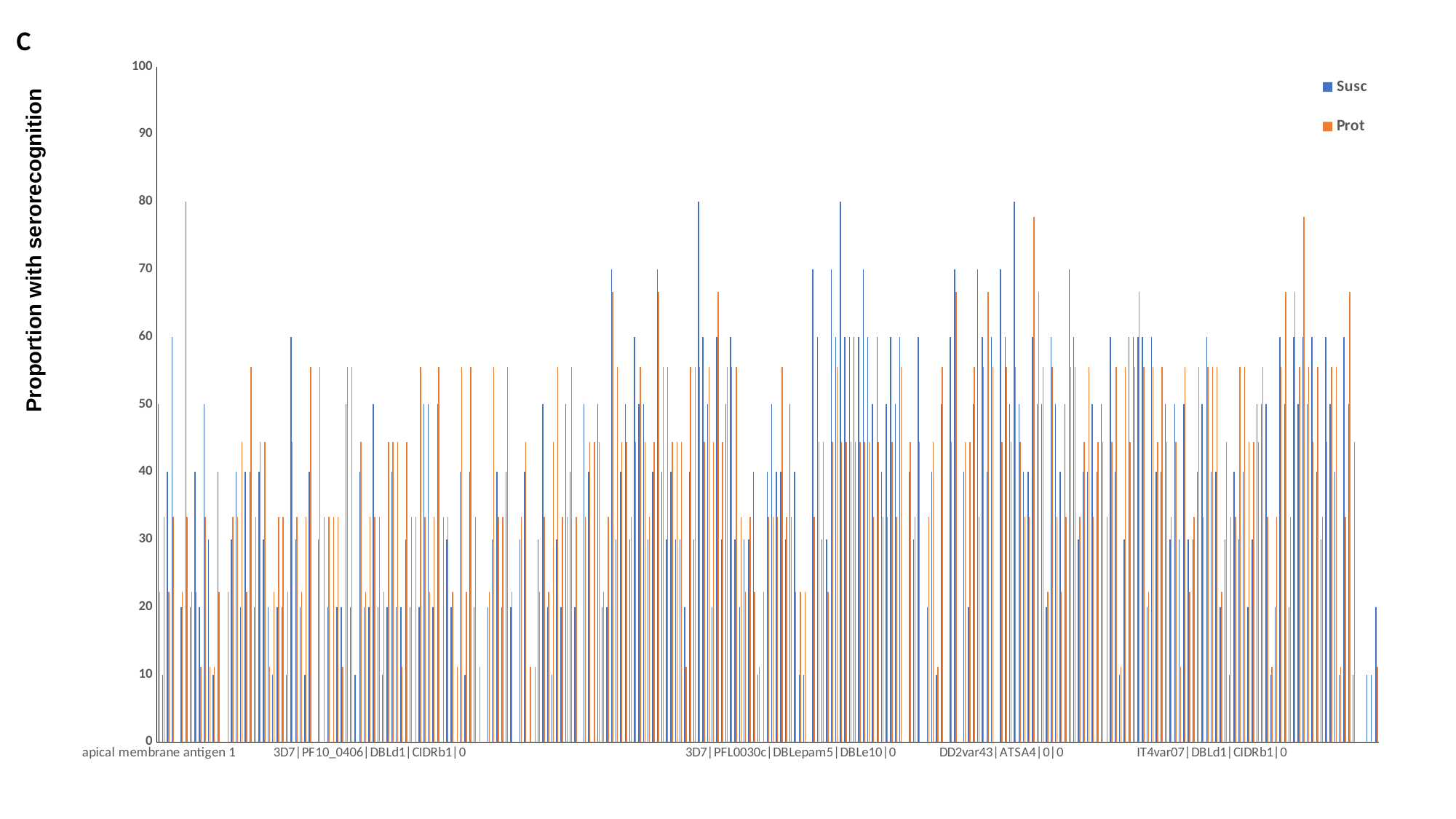

C
### Chart
| Category | Susc | Prot |
|---|---|---|
| apical membrane antigen 1 | 50.0 | 22.22222222222222 |
| circumsporozoite (CS) protein | 10.0 | 33.333333333333336 |
| merozoite surface protein 1 | 40.0 | 22.22222222222222 |
| merozoite surface protein 1 | 60.0 | 33.333333333333336 |
| | 0.0 | 0.0 |
| 3D7|PF11_0008|DBLalpha tag Group A | 20.0 | 22.22222222222222 |
| 3D7|PF13_0003|DBLalpha tag Group A | 80.0 | 33.333333333333336 |
| 3D7|PFA0015c|DBLalpha tag Group A | 20.0 | 22.22222222222222 |
| 3D7|MAL6P1.4|DBLalpha tag Group B/A | 40.0 | 22.22222222222222 |
| 3D7|MAL6P1.252|DBLalpha tag Group C | 20.0 | 11.11111111111111 |
| 3D7|PF07_0049|DBLalpha tags | 50.0 | 33.333333333333336 |
| 3D7|PF08_0106|DBLalpha tags | 30.0 | 11.11111111111111 |
| 3D7|PFB0010w|DBLalpha tags | 10.0 | 11.11111111111111 |
| 3D7|PFL1960w|DBLalpha tags | 40.0 | 22.22222222222222 |
| | 0.0 | 0.0 |
| 3D7|MAL6P1.1|DBLa0.8|CIDRa4|0 | 0.0 | 22.22222222222222 |
| 3D7|MAL6P1.252|DBLa0.21|CIDRa2.1|0 | 30.0 | 33.333333333333336 |
| 3D7|MAL6P1.252|DBLb4|DBLd1|0 | 40.0 | 33.333333333333336 |
| 3D7|MAL6P1.252|DBLd1|CIDRb1|0 | 20.0 | 44.44444444444444 |
| 3D7|MAL6P1.4|DBLa0.6|CIDRa3.2|0 | 40.0 | 22.22222222222222 |
| 3D7|MAL6P1.4|DBLb5|DBLg13|0 | 40.0 | 55.55555555555556 |
| 3D7|MAL6P1.4|DBLd4|CIDRg1|0 | 20.0 | 33.333333333333336 |
| 3D7|MAL6P1.4|DBLe2|DBLe7|0 | 40.0 | 44.44444444444444 |
| 3D7|MAL6P1.4|DBLe7|DBLe3|0 | 30.0 | 44.44444444444444 |
| 3D7|MAL7P1.212|DBLa0.17|CIDRa3.1|0 | 20.0 | 11.11111111111111 |
| 3D7|MAL7P1.50|DBLd1|CIDRg10|0 | 10.0 | 22.22222222222222 |
| 3D7|MAL7P1.55|DBLa0.9|CIDRa2.4|0 | 20.0 | 33.333333333333336 |
| 3D7|MAL7P1.55|DBLd1|CIDRg5|0 | 20.0 | 33.333333333333336 |
| 3D7|MAL8P1.220|DBLd1|CIDRb1|0 | 10.0 | 22.22222222222222 |
| 3D7|PF07_0048|DBLd1|CIDRb6|0 | 60.0 | 44.44444444444444 |
| 3D7|PF07_0049|DBLa0.17|CIDRa3.1|0 | 30.0 | 33.333333333333336 |
| 3D7|PF07_0049|DBLd1|CIDRg7|0 | 20.0 | 22.22222222222222 |
| 3D7|PF07_0050|DBLa0.18|CIDRa6|0 | 10.0 | 33.333333333333336 |
| 3D7|PF07_0050|DBLb6|DBLg9|0 | 40.0 | 55.55555555555556 |
| 3D7|PF07_0051|DBLa0.1|CIDRa3.1|0 | 0.0 | 0.0 |
| 3D7|PF07_0051|DBLd1|CIDRb1|0 | 30.0 | 55.55555555555556 |
| 3D7|PF07_0139|DBLa0.16|CIDRa3.4|0 | 0.0 | 33.333333333333336 |
| 3D7|PF07_0139|DBLd1|CIDRb1|0 | 20.0 | 33.333333333333336 |
| 3D7|PF07_0139|DBLe4|ATSB1|0 | 0.0 | 33.333333333333336 |
| 3D7|PF08_0103|DBLa0.12|CIDRa2.2|0 | 20.0 | 33.333333333333336 |
| 3D7|PF08_0106|DBLa0.2|CIDRa3.1|0 | 20.0 | 11.11111111111111 |
| 3D7|PF08_0106|DBLd1|CIDRb1|0 | 50.0 | 55.55555555555556 |
| 3D7|PF08_0107|DBLd1|CIDRg2|0 | 20.0 | 55.55555555555556 |
| 3D7|PF08_0142|DBLa0.9|CIDRa2.11|0 | 10.0 | 0.0 |
| 3D7|PF10_0001|DBLd1|CIDRg4|0 | 40.0 | 44.44444444444444 |
| 3D7|PF10_0406|DBLa0.9|CIDRa2.7|0 | 20.0 | 22.22222222222222 |
| 3D7|PF10_0406|DBLd1|CIDRb1|0 | 20.0 | 33.333333333333336 |
| 3D7|PF11_0007|DBLa0.15|CIDRa3.2|0 | 50.0 | 33.333333333333336 |
| 3D7|PF11_0007|DBLd1|CIDRb1|0 | 20.0 | 33.333333333333336 |
| 3D7|PF13_0001|DBLa0.11|CIDRa2.4|0 | 10.0 | 22.22222222222222 |
| 3D7|PF13_0001|DBLd1|CIDRb1|0 | 20.0 | 44.44444444444444 |
| 3D7|PF13_0364|DBLa0.16|CIDRa3.4|0 | 40.0 | 44.44444444444444 |
| 3D7|PF13_0364|DBLd1|CIDRb6|0 | 20.0 | 44.44444444444444 |
| 3D7|PFA0005w|DBLa0.11|CIDRa2.8|0 | 20.0 | 11.11111111111111 |
| 3D7|PFA0005w|DBLd1|CIDRb1|0 | 30.0 | 44.44444444444444 |
| 3D7|PFA0765c|DBLa0.5|CIDRa2.8|0 | 20.0 | 33.333333333333336 |
| 3D7|PFB0010w|DBLa0.7|CIDRa2.2|0 | 0.0 | 33.333333333333336 |
| 3D7|PFB0010w|DBLg11|ATSB1|0 | 20.0 | 55.55555555555556 |
| 3D7|PFB1055c|DBLa0.16|CIDRa3.4|0 | 50.0 | 33.333333333333336 |
| 3D7|PFC1120c|DBLa0.9|CIDRa2.1|0 | 50.0 | 22.22222222222222 |
| 3D7|PFC1120c|DBLd1|CIDRb7|0 | 20.0 | 33.333333333333336 |
| 3D7|PFD0005w|DBLg5|DBLd1|0 | 50.0 | 55.55555555555556 |
| 3D7|PFD0615c|DBLa0.17|CIDRa3.1|0 | 0.0 | 33.333333333333336 |
| 3D7|PFD0615c|DBLd1|CIDRb1|0 | 30.0 | 33.333333333333336 |
| 3D7|PFD0625c|DBLa0.1|CIDRa3.2|0 | 20.0 | 22.22222222222222 |
| 3D7|PFD0630c|DBLa0.1|CIDRa3.1|0 | 0.0 | 11.11111111111111 |
| 3D7|PFD0630c|DBLd1|CIDRg2|0 | 40.0 | 55.55555555555556 |
| 3D7|PFD0635c|DBLa0.1|CIDRa3.1|0 | 10.0 | 22.22222222222222 |
| 3D7|PFD0995c|DBLa0.1|CIDRa3.2|0 | 40.0 | 55.55555555555556 |
| 3D7|PFD0995c|DBLd1|CIDRg6|0 | 20.0 | 33.333333333333336 |
| 3D7|PFD1005c|DBLa0.8|CIDRa4|0 | 0.0 | 11.11111111111111 |
| 3D7|PFD1005c|DBLd1|CIDRg11|0 | 0.0 | 0.0 |
| 3D7|PFD1015c|DBLa0.24|CIDRa3.4|0 | 20.0 | 22.22222222222222 |
| 3D7|PFD1015c|DBLd1|CIDRb1|0 | 30.0 | 55.55555555555556 |
| 3D7|PFD1245c|DBLa0.14|CIDRa4|0 | 40.0 | 33.333333333333336 |
| 3D7|PFD1245c|DBLd1|CIDRb1|0 | 20.0 | 33.333333333333336 |
| 3D7|PFE0005w|DBLd1|CIDRb1|0 | 40.0 | 55.55555555555556 |
| 3D7|PFI0005w|DBLd1|CIDRg12|0 | 20.0 | 22.22222222222222 |
| 3D7|PFL0005w|DBLa0.9|CIDRa2.2|0 | 0.0 | 0.0 |
| 3D7|PFL0005w|DBLd1|CIDRb1|0 | 30.0 | 33.333333333333336 |
| 3D7|PFL0020w|DBLg14|DBLz5|0 | 40.0 | 44.44444444444444 |
| 3D7|PFL0020w|DBLz5|DBLe4|0 | 0.0 | 11.11111111111111 |
| 3D7|PFL0935c|DBLa0.16|CIDRa3.4|0 | 0.0 | 11.11111111111111 |
| 3D7|PFL0935c|DBLd1|CIDRg12|0 | 30.0 | 22.22222222222222 |
| 3D7|PFL1950w|DBLa0.7|CIDRa4|0 | 50.0 | 33.333333333333336 |
| 3D7|PFL1950w|DBLd1|CIDRb1|0 | 20.0 | 22.22222222222222 |
| 3D7|PFL1955w|DBLa0.16|CIDRa3.4|0 | 10.0 | 44.44444444444444 |
| 3D7|PFL1955w|DBLd1|CIDRb1|0 | 30.0 | 55.55555555555556 |
| 3D7|PFL1960w|DBLa0.20|CIDRa3.1|0 | 20.0 | 33.333333333333336 |
| 3D7|PFL1960w|DBLd1|CIDRb1|0 | 50.0 | 33.333333333333336 |
| 3D7|PFL2665c|DBLa0.19|CIDRa2.3|0 | 40.0 | 55.55555555555556 |
| 3D7|PFL2665c|DBLd1|CIDRb1|0 | 20.0 | 33.333333333333336 |
| | 0.0 | 0.0 |
| 3D7|MAL6P1.316|DBLa2|CIDRa1.8|0 | 50.0 | 33.333333333333336 |
| 3D7|MAL6P1.316|DBLb12|DBLg4|0 | 40.0 | 44.44444444444444 |
| 3D7|MAL6P1.316|DBLz3|DBLe12|0 | 0.0 | 44.44444444444444 |
| 3D7|PF08_0140|CIDRa1.6|DBLb12|DBLg4 | 50.0 | 44.44444444444444 |
| 3D7|PF08_0140|DBLa2|CIDRa1.6|0 | 20.0 | 22.22222222222222 |
| 3D7|PF08_0140|DBLa2|CIDRa1.6|DBLb12 | 20.0 | 33.333333333333336 |
| 3D7|PF08_0140|DBLb12|DBLg4|0 | 70.0 | 66.66666666666667 |
| 3D7|PF08_0140|DBLd1|CIDRb1|0 | 30.0 | 55.55555555555556 |
| 3D7|PF08_0141|DBLa1.6|CIDRd2|0 | 40.0 | 44.44444444444444 |
| 3D7|PF08_0141|DBLg14|DBLz5|0 | 50.0 | 44.44444444444444 |
| 3D7|PF08_0141|DBLz5|DBLe4|0 | 30.0 | 33.333333333333336 |
| 3D7|PF08_0141e1s1|PfEMP1 - Extracellular - non-CD36-binding | 60.0 | 44.44444444444444 |
| 3D7|PF08_0141e1s2|PfEMP1 - Extracellular - non-CD36-binding | 50.0 | 55.55555555555556 |
| 3D7|PF08_0141e1s3|PfEMP1 - Extracellular - non-CD36-binding | 50.0 | 44.44444444444444 |
| 3D7|PF11_0008|DBLa1.5|CIDRg3|0 | 30.0 | 33.333333333333336 |
| 3D7|PF11_0008|DBLg12|DBLd5|0 | 40.0 | 44.44444444444444 |
| 3D7|PF11_0008e1s1|PfEMP1 - Extracellular - non-CD36-binding | 70.0 | 66.66666666666667 |
| 3D7|PF11_0008e1s2|PfEMP1 - Extracellular - non-CD36-binding | 40.0 | 55.55555555555556 |
| 3D7|PF11_0008e1s3|PfEMP1 - Extracellular - non-CD36-binding | 30.0 | 55.55555555555556 |
| 3D7|PF11_0521|DBLb3|DBLb6|0 | 40.0 | 44.44444444444444 |
| 3D7|PF11_0521|DBLb6|DBLd3|0 | 30.0 | 44.44444444444444 |
| 3D7|PF13_0003|DBLa1.6|CIDRd1|0 | 30.0 | 44.44444444444444 |
| 3D7|PF13_0003|DBLb3|DBLg12|0 | 20.0 | 11.11111111111111 |
| 3D7|PF13_0003|DBLd5|CIDRb3|0 | 40.0 | 55.55555555555556 |
| 3D7|PF13_0003|DBLg12|DBLd5|0 | 30.0 | 55.55555555555556 |
| 3D7|PFA0015c|DBLa1.3|DBLe8|0 | 80.0 | 55.55555555555556 |
| 3D7|PFD0020c|DBLa1.2|CIDRa1.1|0 | 60.0 | 44.44444444444444 |
| 3D7|PFD0020c|DBLb12|DBLg6|0 | 50.0 | 55.55555555555556 |
| 3D7|PFD0020c|DBLd1|CIDRg8|0 | 20.0 | 44.44444444444444 |
| 3D7|PFD0020c|DBLg6|DBLg11|0 | 60.0 | 66.66666666666667 |
| 3D7|PFD1235w|DBLa1.4|CIDRa1.6|0 | 30.0 | 44.44444444444444 |
| 3D7|PFD1235w|DBLb3|DBLb3|0 | 50.0 | 55.55555555555556 |
| 3D7|PFD1235w|DBLb3|DBLg13|0 | 60.0 | 55.55555555555556 |
| 3D7|PFD1235w|DBLd1|CIDRb5|0 | 30.0 | 55.55555555555556 |
| 3D7|PFD1235w|DBLg13|DBLd1|0 | 20.0 | 33.333333333333336 |
| 3D7|PFE1640w|DBLb1|DBLg15|0 | 30.0 | 22.22222222222222 |
| 3D7|PFE1640w|DBLe1|DBLg8|0 | 30.0 | 33.333333333333336 |
| 3D7|PFE1640w|DBLg15|DBLe1|0 | 40.0 | 22.22222222222222 |
| 3D7|PFE1640w|DBLg8|DBLz1|0 | 10.0 | 11.11111111111111 |
| 3D7|PFE1640w|DBLz1|DBLe5|0 | 0.0 | 22.22222222222222 |
| 3D7|PFF0020c|DBLa1.3|DBLe8|0 | 40.0 | 33.333333333333336 |
| 3D7|PFI1820w|DBLa1.3|DBLe8|0 | 50.0 | 33.333333333333336 |
| 3D7|PFL0020w|DBLa0.18|CIDRa5|0 | 40.0 | 33.333333333333336 |
| 3D7|PFL0020w|DBLb5|DBLg14|0 | 40.0 | 55.55555555555556 |
| 3D7|PFL0030c|DBLepam4|DBLepam5|0 | 30.0 | 33.333333333333336 |
| 3D7|PFL0030c|DBLepam5|DBLe10|0 | 50.0 | 33.333333333333336 |
| 3D7|PFL0030c|DBLpam1|DBLpam2|0 | 40.0 | 22.22222222222222 |
| 3D7|PFL0030c|DBLpam2|CIDRpam|0 | 10.0 | 22.22222222222222 |
| 3D7|PFL0030c|DBLpam3|DBLepam4|0 | 10.0 | 22.22222222222222 |
| | 0.0 | 0.0 |
| 3D7|MAL6P1.252-e2|PfEMP1 - Intracellular | 70.0 | 33.333333333333336 |
| 3D7|MAL7P1.55|ATSB13|0|0 | 60.0 | 44.44444444444444 |
| 3D7|MAL8P1.220|ATSB1|0|0 | 30.0 | 44.44444444444444 |
| 3D7|PF07_0048|ATSB7|0|0 | 30.0 | 22.22222222222222 |
| 3D7|PF07_0048e2s1|PfEMP1 - Intracellular | 70.0 | 44.44444444444444 |
| 3D7|PF08_0107|ATSB2|0|0 | 60.0 | 55.55555555555556 |
| 3D7|PF08_0140e2s1|PfEMP1 - Intracellular | 80.0 | 44.44444444444444 |
| 3D7|PF08_0141|ATSA3|0|0 | 60.0 | 44.44444444444444 |
| 3D7|PF08_0141e2s1|PfEMP1 - Intracellular | 60.0 | 44.44444444444444 |
| 3D7|PF10_0001|ATSB1|0|0 | 60.0 | 44.44444444444444 |
| 3D7|PF10_0406|ATSB1|0|0 | 60.0 | 44.44444444444444 |
| 3D7|PF11_0008e2s1|PfEMP1 - Intracellular | 70.0 | 44.44444444444444 |
| 3D7|PF11_0521|ATSA2|0|0 | 60.0 | 44.44444444444444 |
| 3D7|PF11_0521|PfEMP1 - Intracellular | 50.0 | 33.333333333333336 |
| 3D7|PF13_0003|ATSA2|0|0 | 60.0 | 44.44444444444444 |
| 3D7|PFC0005w|ATSB1|0|0 | 40.0 | 33.333333333333336 |
| 3D7|PFD0995ce2s1|PfEMP1 - Intracellular | 50.0 | 33.333333333333336 |
| 3D7|PFD1000c|ATSB19|0|0 | 60.0 | 44.44444444444444 |
| 3D7|PFD1235w|ATSA2|0|0 | 50.0 | 33.333333333333336 |
| 3D7|PFL1955w|ATSB7|0|0 | 60.0 | 55.55555555555556 |
| | 0.0 | 0.0 |
| 3D7|AMA1 | 40.0 | 44.44444444444444 |
| 3D7|MSP1 - segment 1 | 30.0 | 33.333333333333336 |
| 3D7|MSP1 - segment 2 | 60.0 | 44.44444444444444 |
| | 0.0 | 0.0 |
| 3D7|PF07_0049 | 20.0 | 33.333333333333336 |
| 3D7|PF08_0140 | 40.0 | 44.44444444444444 |
| 3D7|PF11_0007 | 10.0 | 11.11111111111111 |
| 3D7|PF11_0521PF11_0521_1|DBL|CIDR|0 | 50.0 | 55.55555555555556 |
| | 0.0 | 0.0 |
| 3D7|rifin||| | 60.0 | 44.44444444444444 |
| 3D7|rifin||| | 70.0 | 66.66666666666667 |
| | 0.0 | 0.0 |
| 3D7|stevor||| | 40.0 | 44.44444444444444 |
| 3D7|stevor||| | 20.0 | 44.44444444444444 |
| 3D7|stevor||| | 50.0 | 55.55555555555556 |
| 3D7|stevor-1||| | 70.0 | 33.333333333333336 |
| 3D7|stevor-1||| | 60.0 | 55.55555555555556 |
| 3D7|stevor-2||| | 40.0 | 66.66666666666667 |
| 3D7|stevor-2||| | 60.0 | 55.55555555555556 |
| | 0.0 | 0.0 |
| DD2var43|ATSA4|0|0 | 70.0 | 44.44444444444444 |
| DD2var43|CIDRa1.5|DBLg17|0 | 60.0 | 55.55555555555556 |
| DD2var43|CIDRb3|DBLb7|0 | 50.0 | 44.44444444444444 |
| DD2var43|DBLa1.2|CIDRa1.5|0 | 80.0 | 55.55555555555556 |
| DD2var43|DBLb7|DBLg9|0 | 50.0 | 44.44444444444444 |
| DD2var43|DBLd5|CIDRb3|0 | 40.0 | 33.333333333333336 |
| DD2var43|DBLg17|DBLd5|0 | 40.0 | 33.333333333333336 |
| DD2var49|CIDRa1.7|DBLb3|0 | 60.0 | 77.77777777777777 |
| DD2var49|CIDRg2|DBLg17|0 | 50.0 | 66.66666666666667 |
| DD2var49|DBLb3|DBLg11|0 | 50.0 | 55.55555555555556 |
| DD2var49|DBLg17|DBLz4|0 | 20.0 | 22.22222222222222 |
| DD2var49|NTSA8|DBLa1.2|CIDRa1.7 | 60.0 | 55.55555555555556 |
| HB3var03|CIDRa1.4|DBLb3|0 | 50.0 | 33.333333333333336 |
| HB3var03|CIDRb3|DBLb7|0 | 40.0 | 22.22222222222222 |
| HB3var03|DBLb3|DBLg12|0 | 50.0 | 33.333333333333336 |
| HB3var05|ATSA2|0|0 | 70.0 | 55.55555555555556 |
| HB3var1csa|ATS|0|0 | 60.0 | 55.55555555555556 |
| HB3var1csa|CIDRa|DBLb|0 | 30.0 | 33.333333333333336 |
| HB3var1csa|DBLa|CIDRa|0 | 40.0 | 44.44444444444444 |
| HB3var1csa|DBLb|DBLg|0 | 40.0 | 55.55555555555556 |
| HB3var1csa|DBLe|DBLg|0 | 50.0 | 33.333333333333336 |
| HB3var1csa|DBLg|DBLe|0 | 40.0 | 44.44444444444444 |
| HB3var1csa|DBLg|DBLz|0 | 50.0 | 44.44444444444444 |
| HB3var1csa|DBLz|DBLe|0 | 0.0 | 33.333333333333336 |
| IGHvar14|ATS|0|0 | 60.0 | 44.44444444444444 |
| IGHvar14|CIDRa|DBLb|0 | 40.0 | 55.55555555555556 |
| IGHvar14|DBLa|CIDRa|0 | 10.0 | 11.11111111111111 |
| IGHvar14|DBLb|DBLg|0 | 30.0 | 55.55555555555556 |
| IGHvar14|DBLe|DBLe|0 | 60.0 | 44.44444444444444 |
| IGHvar14|DBLg|DBLe|0 | 60.0 | 55.55555555555556 |
| IGHvar23|ATS|0|0 | 60.0 | 66.66666666666667 |
| IGHvar23|CIDRa|DBLb|0 | 60.0 | 55.55555555555556 |
| IGHvar23|DBLa|CIDRa|0 | 20.0 | 22.22222222222222 |
| IGHvar23|DBLb|DBLg|0 | 60.0 | 55.55555555555556 |
| IGHvar23|DBLd|CIDRb|0 | 40.0 | 44.44444444444444 |
| IGHvar23|DBLg|DBLd|0 | 40.0 | 55.55555555555556 |
| IGHvar23|DBLg|DBLg|0 | 50.0 | 44.44444444444444 |
| IT4var06|ATS|0|0 | 30.0 | 33.333333333333336 |
| IT4var06|CIDRa|DBLb|0 | 50.0 | 44.44444444444444 |
| IT4var06|DBLa|CIDRa|0 | 30.0 | 11.11111111111111 |
| IT4var06|DBLb|DBLg|0 | 50.0 | 55.55555555555556 |
| IT4var06|DBLd|0|0 | 30.0 | 22.22222222222222 |
| IT4var06|DBLd|CIDRb|0 | 30.0 | 33.333333333333336 |
| IT4var07|CIDRa1.4|DBLb1|0 | 40.0 | 55.55555555555556 |
| IT4var07|DBLb1|DBLb3|0 | 50.0 | 33.333333333333336 |
| IT4var07|DBLb3|DBLg10|0 | 60.0 | 55.55555555555556 |
| IT4var07|DBLd1|CIDRb1|0 | 40.0 | 55.55555555555556 |
| IT4var07|NTSA6|DBLa1.7|CIDRa1.4 | 40.0 | 55.55555555555556 |
| IT4var19|ATSB3|0|0 | 20.0 | 22.22222222222222 |
| IT4var19|CIDRa1.1|DBLb12|0 | 30.0 | 44.44444444444444 |
| IT4var19|CIDRb1|DBLg9|0 | 10.0 | 33.333333333333336 |
| IT4var19|DBLb12|DBLg6|0 | 40.0 | 33.333333333333336 |
| IT4var19|DBLd1|CIDRb1|0 | 30.0 | 55.55555555555556 |
| IT4var19|DBLg6|DBLd1|0 | 40.0 | 55.55555555555556 |
| IT4var20|ATSB16|0|0 | 20.0 | 44.44444444444444 |
| IT4var20|CIDRa1.1|DBLb12|0 | 30.0 | 44.44444444444444 |
| IT4var20|DBLb12|DBLg6|0 | 50.0 | 44.44444444444444 |
| IT4var20|DBLd1|CIDRb1|0 | 50.0 | 55.55555555555556 |
| IT4var20|DBLg6|DBLd1|0 | 50.0 | 33.333333333333336 |
| IT4var20|NTSB3|DBLa2|CIDRa1.1 | 10.0 | 11.11111111111111 |
| IT4var22|CIDRa1.7|DBLb3|0 | 20.0 | 33.333333333333336 |
| IT4var22|DBLa1.4|CIDRa1.7|0 | 60.0 | 55.55555555555556 |
| IT4var22|DBLb3|DBLg10|0 | 50.0 | 66.66666666666667 |
| IT4var22|DBLd1|CIDRb1|0 | 20.0 | 33.333333333333336 |
| IT4var22|DBLg10|DBLg11|0 | 60.0 | 66.66666666666667 |
| IT4var22|DBLg11|DBLd1|0 | 50.0 | 55.55555555555556 |
| IT4var22|NTSA2|DBLa1.4|CIDRa1.7 | 60.0 | 77.77777777777777 |
| MaliRF327|CIDRa|DBLb|0 | 50.0 | 55.55555555555556 |
| MaliRF327|DBLa|CIDRa|0 | 60.0 | 44.44444444444444 |
| MaliRF327|DBLb|DBLg|0 | 40.0 | 55.55555555555556 |
| MaliRF327|DBLd|CIDRb|0 | 30.0 | 33.333333333333336 |
| MaliRF327|DBLg|DBLd|0 | 60.0 | 44.44444444444444 |
| MaliRF327|DBLg|DBLg|0 | 50.0 | 55.55555555555556 |
| MaliRF398|CIDRa|DBLb|0 | 40.0 | 55.55555555555556 |
| MaliRF398|DBLa|CIDRa|0 | 10.0 | 11.11111111111111 |
| MaliRF398|DBLb|DBLg|0 | 60.0 | 33.333333333333336 |
| MaliRF398|DBLg|DBLz|0 | 50.0 | 66.66666666666667 |
| MaliRF398|DBLz|DBLe|0 | 10.0 | 44.44444444444444 |
| | 0.0 | 0.0 |
| | 0.0 | 0.0 |
| Tetanus toxin_0.01 | 10.0 | 0.0 |
| Tetanus toxin_0.03 | 10.0 | 0.0 |
| Tetanus toxin_0.1 | 20.0 | 11.11111111111111 |Proportion with serorecognition

## Slide 10
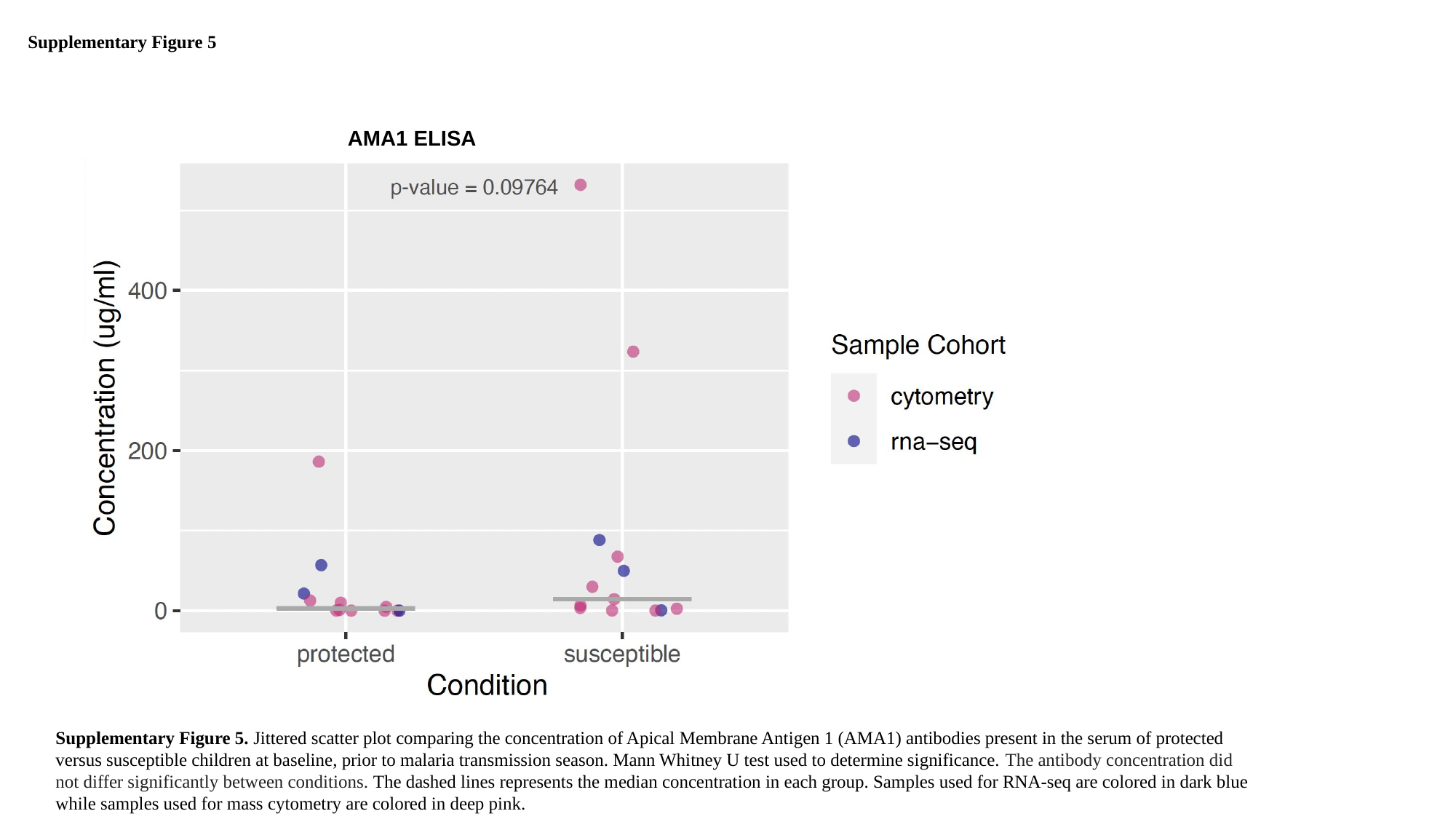

# Supplementary Figure 5
AMA1 ELISA
Supplementary Figure 5. Jittered scatter plot comparing the concentration of Apical Membrane Antigen 1 (AMA1) antibodies present in the serum of protected versus susceptible children at baseline, prior to malaria transmission season. Mann Whitney U test used to determine significance. The antibody concentration did not differ significantly between conditions. The dashed lines represents the median concentration in each group. Samples used for RNA-seq are colored in dark blue while samples used for mass cytometry are colored in deep pink.

## Slide 11
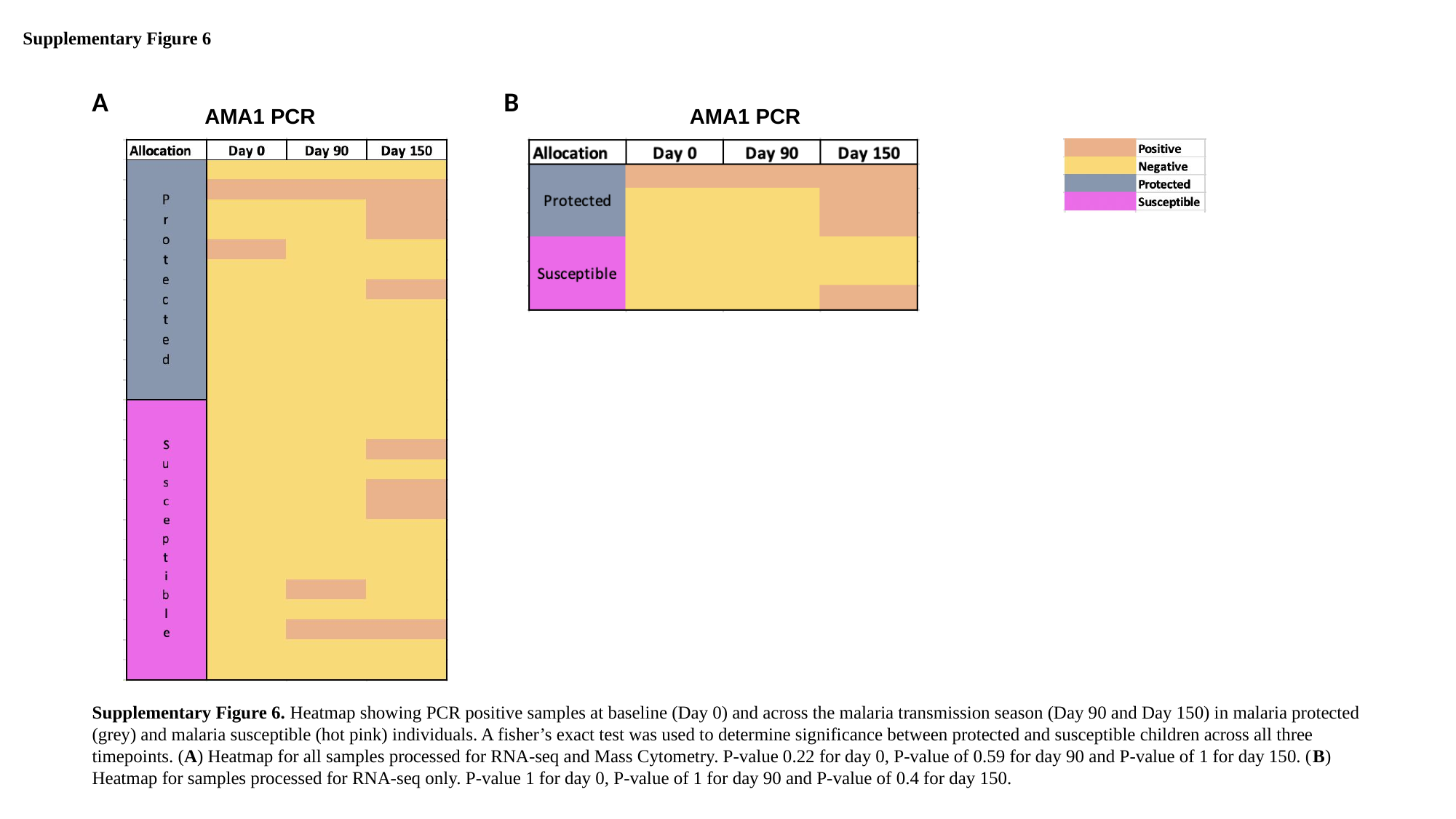

# Supplementary Figure 6
B
A
AMA1 PCR
AMA1 PCR
Supplementary Figure 6. Heatmap showing PCR positive samples at baseline (Day 0) and across the malaria transmission season (Day 90 and Day 150) in malaria protected (grey) and malaria susceptible (hot pink) individuals. A fisher’s exact test was used to determine significance between protected and susceptible children across all three timepoints. (A) Heatmap for all samples processed for RNA-seq and Mass Cytometry. P-value 0.22 for day 0, P-value of 0.59 for day 90 and P-value of 1 for day 150. (B) Heatmap for samples processed for RNA-seq only. P-value 1 for day 0, P-value of 1 for day 90 and P-value of 0.4 for day 150.

## Slide 12
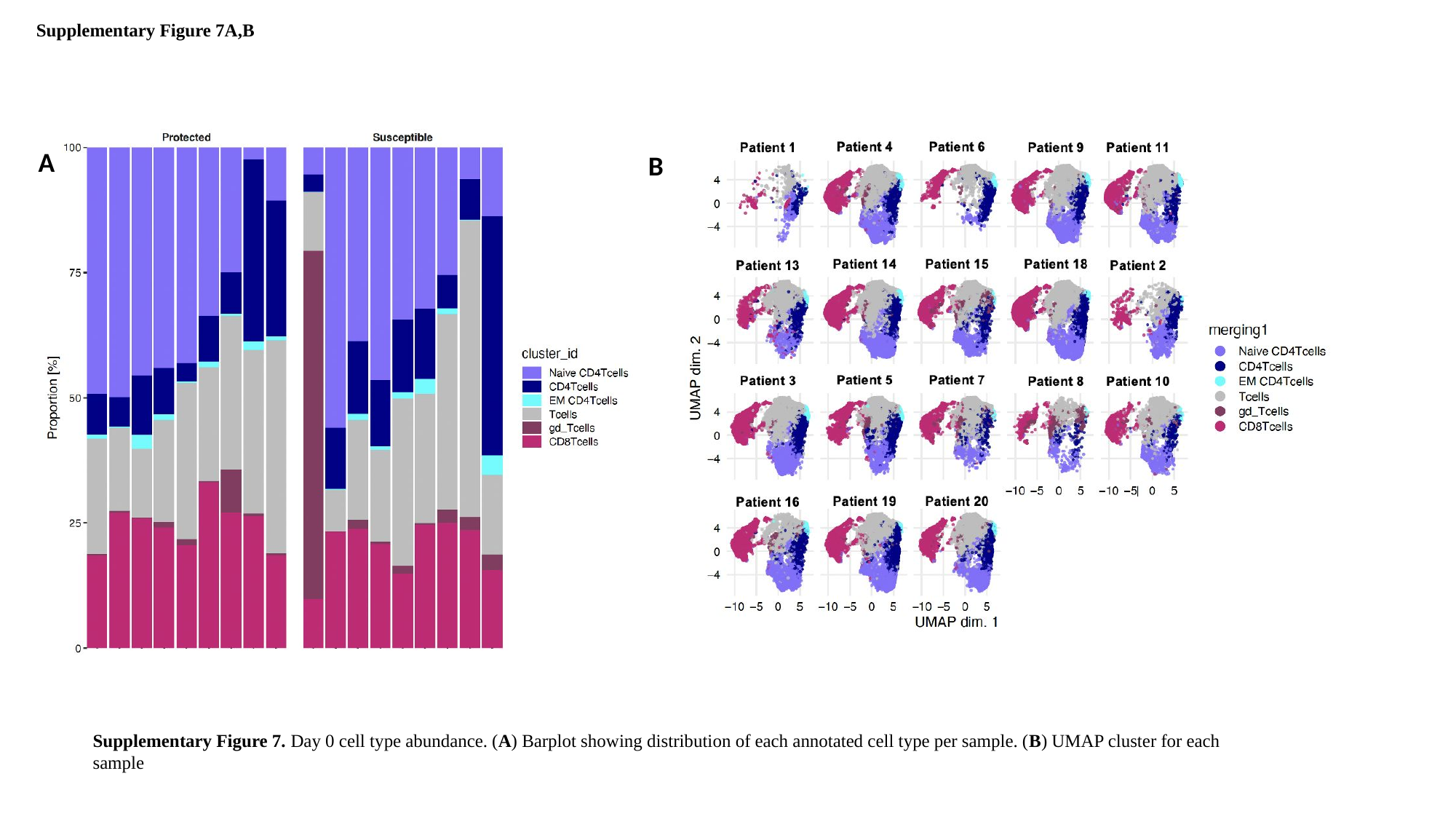

# Supplementary Figure 7A,B
A
B
Supplementary Figure 7. Day 0 cell type abundance. (A) Barplot showing distribution of each annotated cell type per sample. (B) UMAP cluster for each sample

## Slide 13
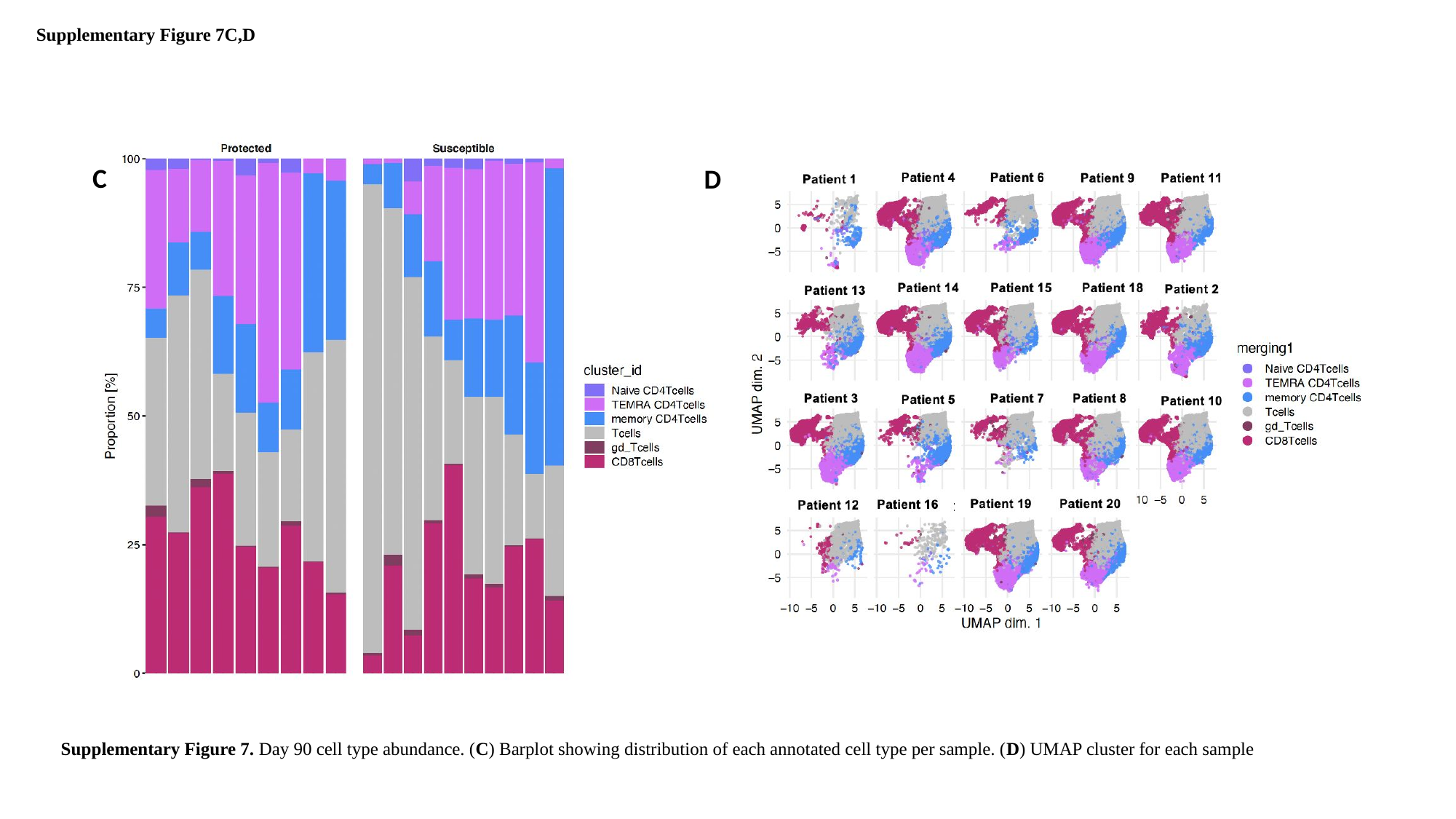

# Supplementary Figure 7C,D
C
D
Supplementary Figure 7. Day 90 cell type abundance. (C) Barplot showing distribution of each annotated cell type per sample. (D) UMAP cluster for each sample

## Slide 14
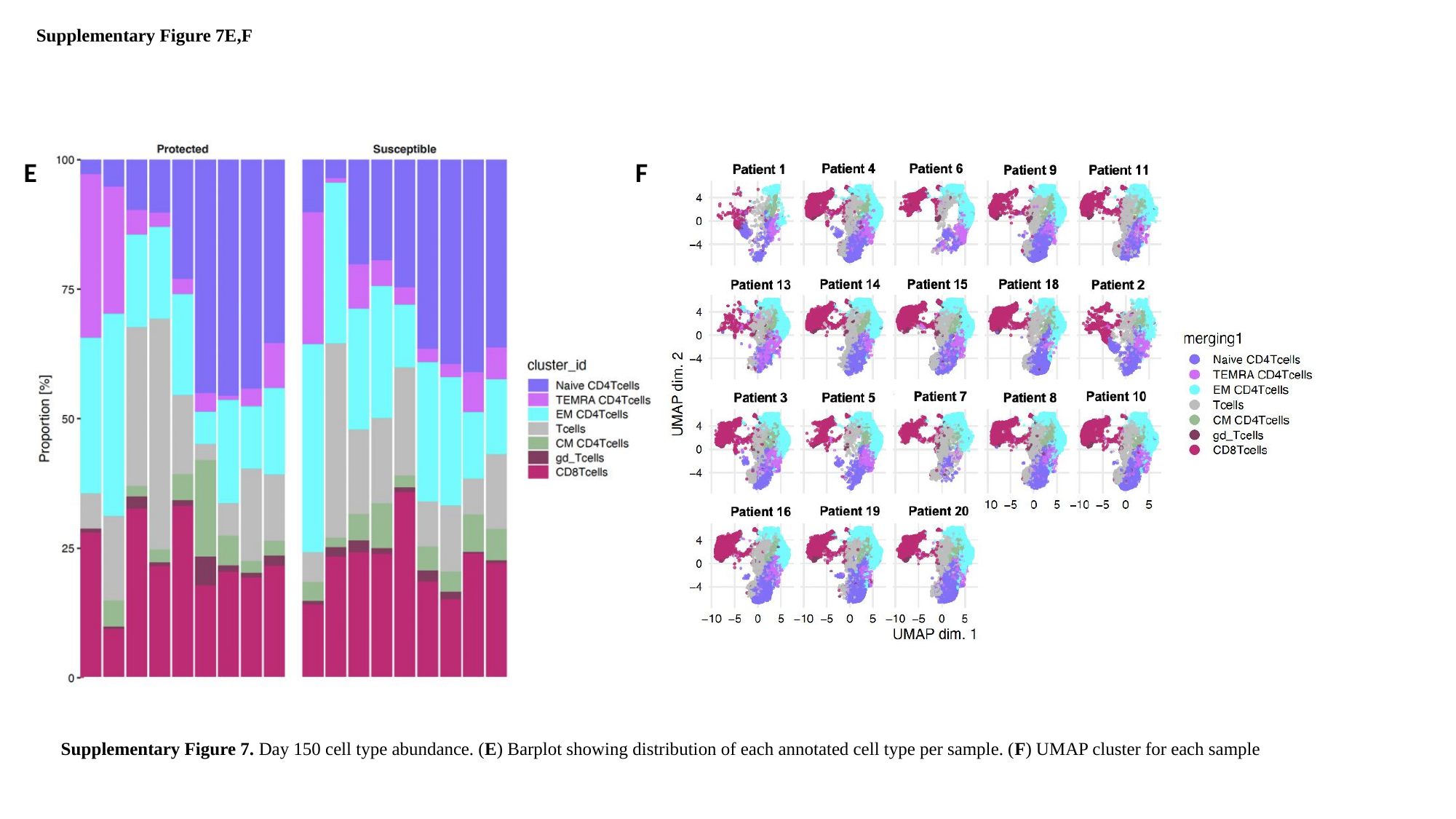

# Supplementary Figure 7E,F
E
F
Supplementary Figure 7. Day 150 cell type abundance. (E) Barplot showing distribution of each annotated cell type per sample. (F) UMAP cluster for each sample

## Slide 15
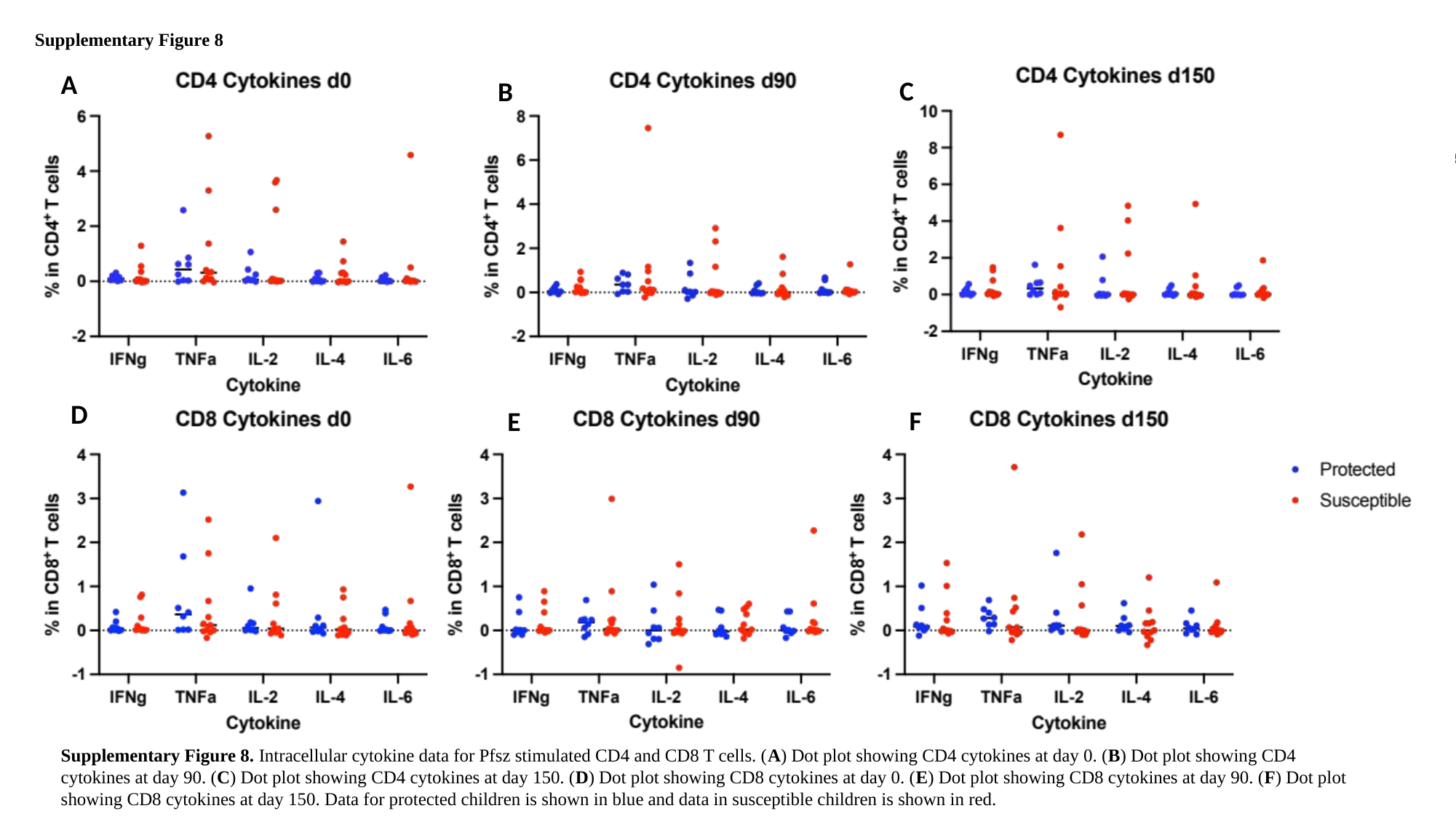

Supplementary Figure 8
A
C
B
D
F
E
Supplementary Figure 8. Intracellular cytokine data for Pfsz stimulated CD4 and CD8 T cells. (A) Dot plot showing CD4 cytokines at day 0. (B) Dot plot showing CD4 cytokines at day 90. (C) Dot plot showing CD4 cytokines at day 150. (D) Dot plot showing CD8 cytokines at day 0. (E) Dot plot showing CD8 cytokines at day 90. (F) Dot plot showing CD8 cytokines at day 150. Data for protected children is shown in blue and data in susceptible children is shown in red.

## Slide 16
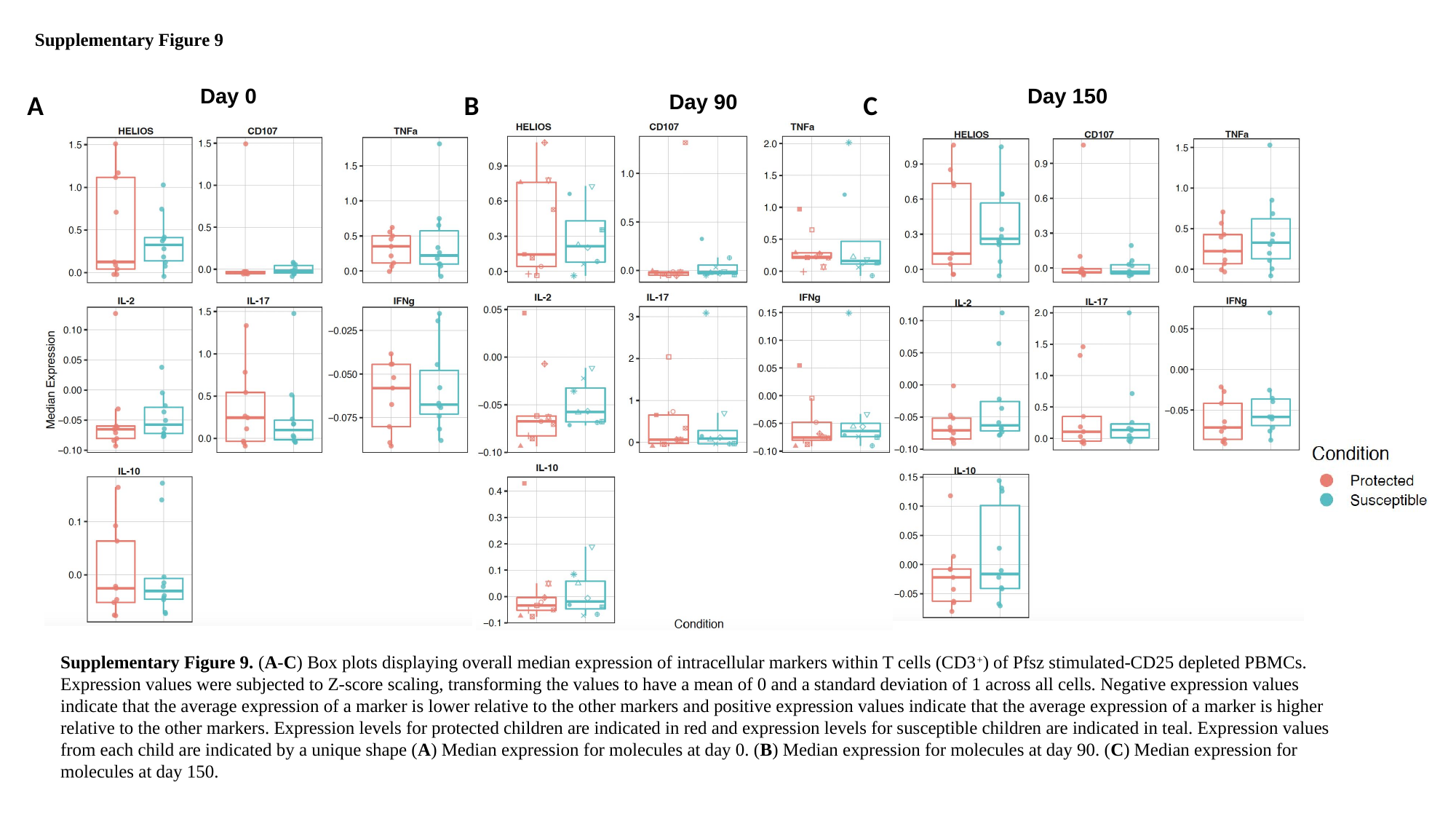

Supplementary Figure 9
Day 0
Day 150
A
B
Day 90
C
Supplementary Figure 9. (A-C) Box plots displaying overall median expression of intracellular markers within T cells (CD3+) of Pfsz stimulated-CD25 depleted PBMCs. Expression values were subjected to Z-score scaling, transforming the values to have a mean of 0 and a standard deviation of 1 across all cells. Negative expression values indicate that the average expression of a marker is lower relative to the other markers and positive expression values indicate that the average expression of a marker is higher relative to the other markers. Expression levels for protected children are indicated in red and expression levels for susceptible children are indicated in teal. Expression values from each child are indicated by a unique shape (A) Median expression for molecules at day 0. (B) Median expression for molecules at day 90. (C) Median expression for molecules at day 150.

## Slide 17
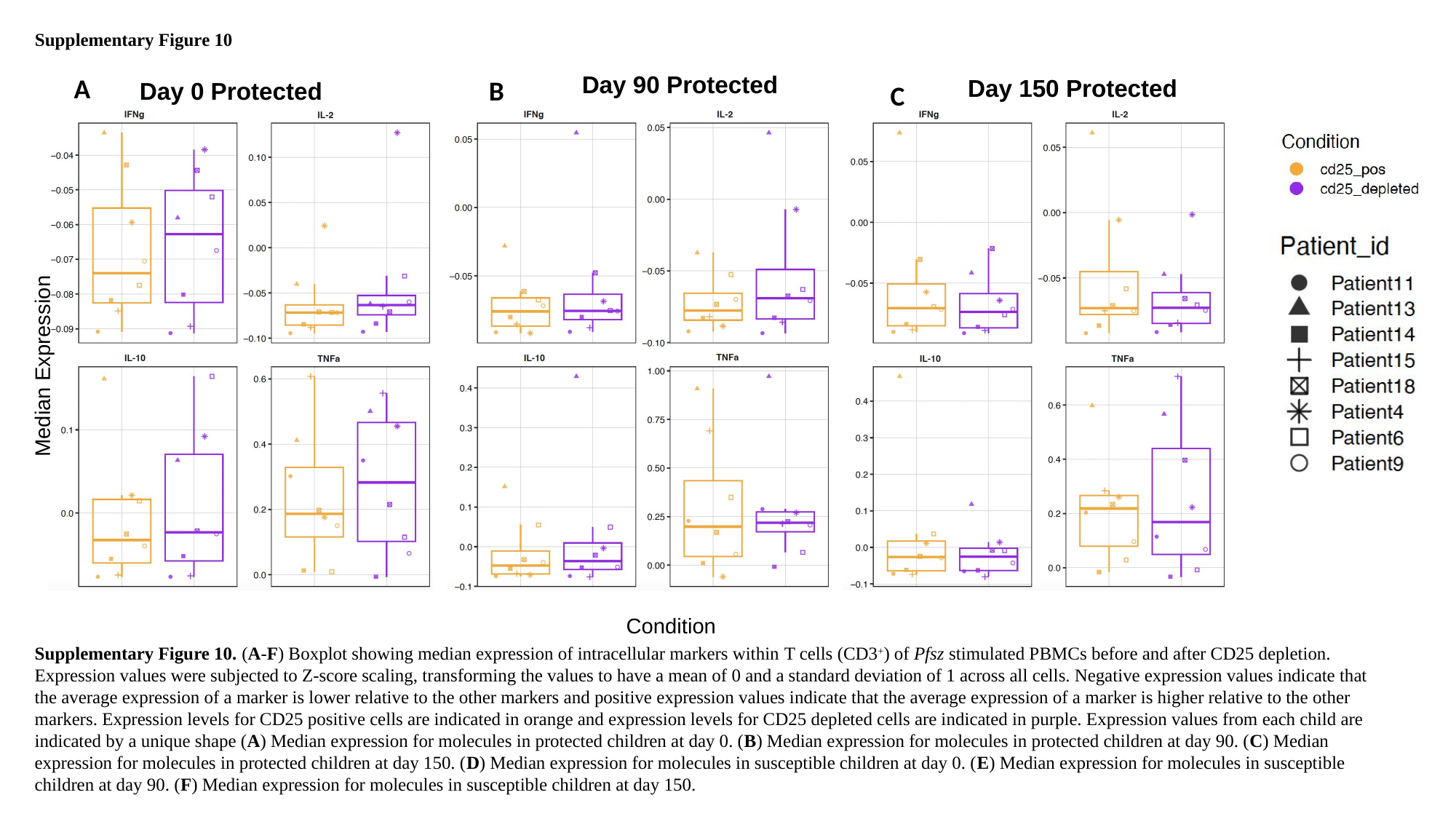

Supplementary Figure 10
Day 90 Protected
A
Day 150 Protected
B
Day 0 Protected
C
Median Expression
Condition
Supplementary Figure 10. (A-F) Boxplot showing median expression of intracellular markers within T cells (CD3+) of Pfsz stimulated PBMCs before and after CD25 depletion. Expression values were subjected to Z-score scaling, transforming the values to have a mean of 0 and a standard deviation of 1 across all cells. Negative expression values indicate that the average expression of a marker is lower relative to the other markers and positive expression values indicate that the average expression of a marker is higher relative to the other markers. Expression levels for CD25 positive cells are indicated in orange and expression levels for CD25 depleted cells are indicated in purple. Expression values from each child are indicated by a unique shape (A) Median expression for molecules in protected children at day 0. (B) Median expression for molecules in protected children at day 90. (C) Median expression for molecules in protected children at day 150. (D) Median expression for molecules in susceptible children at day 0. (E) Median expression for molecules in susceptible children at day 90. (F) Median expression for molecules in susceptible children at day 150.

## Slide 18
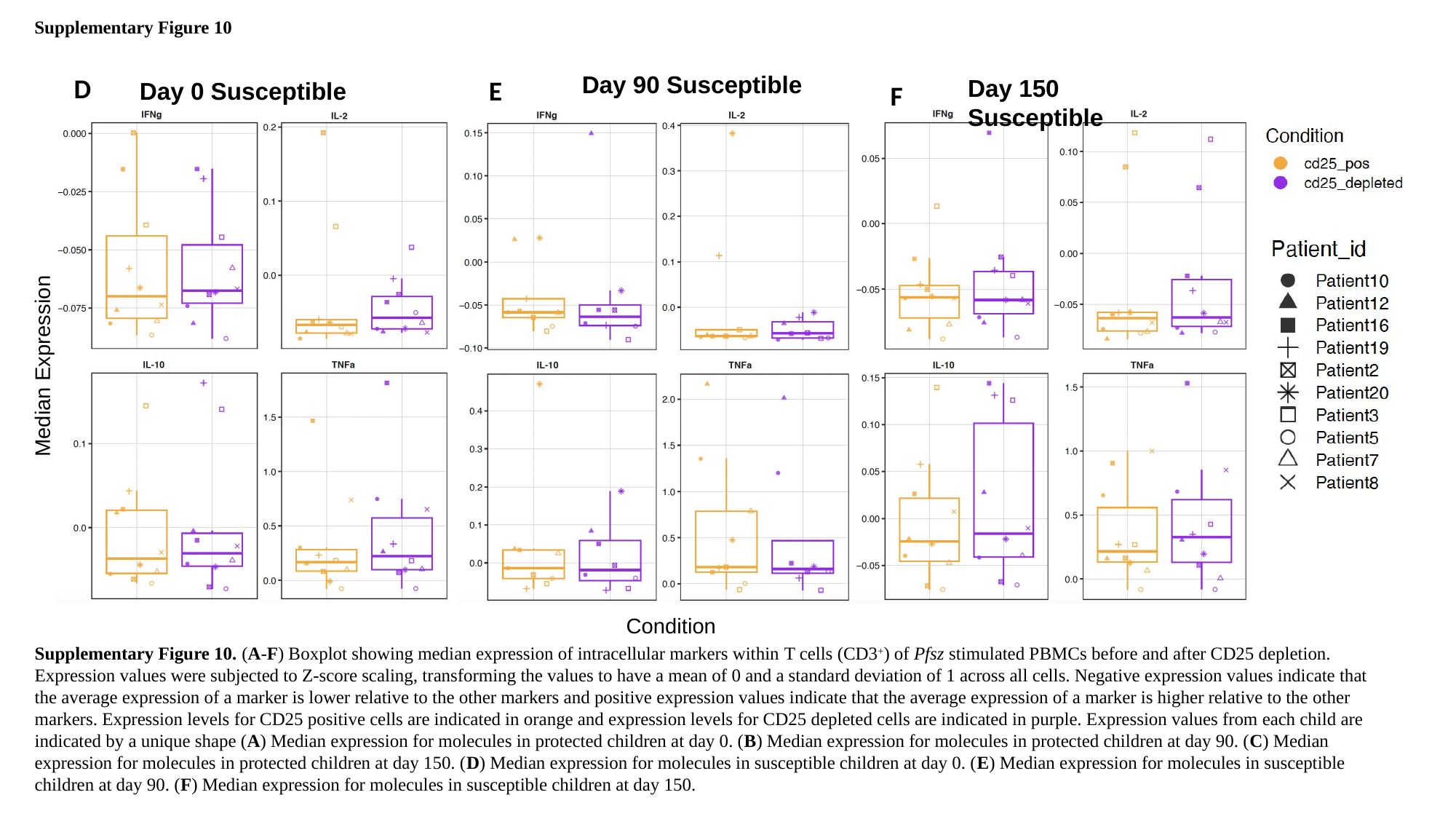

Supplementary Figure 10
Day 90 Susceptible
D
Day 150 Susceptible
E
Day 0 Susceptible
F
Median Expression
Condition
Supplementary Figure 10. (A-F) Boxplot showing median expression of intracellular markers within T cells (CD3+) of Pfsz stimulated PBMCs before and after CD25 depletion. Expression values were subjected to Z-score scaling, transforming the values to have a mean of 0 and a standard deviation of 1 across all cells. Negative expression values indicate that the average expression of a marker is lower relative to the other markers and positive expression values indicate that the average expression of a marker is higher relative to the other markers. Expression levels for CD25 positive cells are indicated in orange and expression levels for CD25 depleted cells are indicated in purple. Expression values from each child are indicated by a unique shape (A) Median expression for molecules in protected children at day 0. (B) Median expression for molecules in protected children at day 90. (C) Median expression for molecules in protected children at day 150. (D) Median expression for molecules in susceptible children at day 0. (E) Median expression for molecules in susceptible children at day 90. (F) Median expression for molecules in susceptible children at day 150.
